# CCAAT/Enhancer-Binding Protein Delta (C/EBPδ): A Previously Unrecognized Tumor Suppressor that Limits the Oncogenic Potential of Pancreatic Ductal Adenocarcinoma Cells

**DOI:** 10.3390/cancers12092546

**Published:** 2020-09-07

**Authors:** Leonie Hartl, JanWillem Duitman, Hella L. Aberson, Kan Chen, Frederike Dijk, Joris J.T.H. Roelofs, Mark P.G. Dings, Gerrit K.J. Hooijer, Pratika Y. Hernanda, Qiunwei Pan, Olivier R. Busch, Marc G.H. Besselink, Ton Boerman, Maikel P. Peppelenbosch, Maarten F. Bijlsma, C. Arnold Spek

**Affiliations:** 1 Laboratory for Experimental Oncology and Radiobiology, Center for Experimental and Molecular Medicine, Amsterdam University Medical Center, University of Amsterdam, 1105 AZ Amsterdam, The Netherlands; j.w.duitman@amsterdamumc.nl (J.D.); h.l.aberson@amsterdamumc.nl (H.L.A.); m.p.dings@amsterdamumc.nl (M.P.G.D.); m.f.bijlsma@amsterdamumc.nl (M.F.B.); c.a.spek@amsterdamumc.nl (C.A.S.); 2 Cancer Center Amsterdam, Amsterdam University Medical Center, University of Amsterdam, 1182 DB Amsterdam, The Netherlands; f.dijk@amc.uva.nl; 3 Department of Gastroenterology and Hepatology, Erasmus Medical Center, University Medical Center Rotterdam, 3015 GD Rotterdam, The Netherlands; chenkan@zstu.edu.cn (K.C.); q.pan@erasmusmc.nl (Q.P.); m.peppelenbosch@erasmusmc.nl (M.P.P.); 4 Department of Pathology, Amsterdam University Medical Center, University of Amsterdam, 1105 AZ Amsterdam, The Netherlands; j.j.roelofs@amsterdamumc.nl (J.J.T.H.R.); g.k.hooijer@amsterdamumc.nl (G.K.J.H.); tonboerman@gmail.com (T.B.); 5 Laboratory of Medical Genetics, University of Wijaya Kusuma Surabaya, Jawa Timur 60225, Indonesia; yuhyi_h@uwks.ac.id; 6 Department of Surgery, Cancer Center Amsterdam, Amsterdam University Medical Center, University of Amsterdam, 1182 DB Amsterdam, The Netherlands; o.r.busch@amsterdamumc.nl; 7 Hepato-Pancreato-Biliary Surgery, Amsterdam University Medical Center, University of Amsterdam, 1105 AZ Amsterdam, The Netherlands; m.g.besselink@amsterdamumc.nl; 8 Oncode Institute, 3521 AL Utrecht Amsterdam, The Netherlands

**Keywords:** CCAAT/enhancer-binding protein delta, CEBPD, pancreatic ductal adenocarcinoma, PDAC, tumor suppressor, ampullary carcinoma, intrapancreatic cholangiocarcinoma

## Abstract

**Simple Summary:**

Here we show that a protein called C/EBPδ is present in healthy pancreas tissue but almost absent in pancreas tumors. Patients with less C/EBPδ in their tumors had the most metastases and the worst survival chances, showing that C/EBPδ has tumor-suppressive properties in pancreatic cancer. In this study, we reactivated C/EBPδ in pancreatic cancer cells *in vitro* and observed a reduction in cell proliferation in a 2-dimentional and 3-dimensional space. This implies that tumor cells grow slower when C/EBPδ is activated and they are likely also less capable to escape the primary tumor in order to form metastases. Conversely, when we deleted C/EBPδ in pancreatic cancer cells, we observed accelerated growth. We suggest that reactivating C/EBPδ can suppress tumor growth and formation of metastases, thereby improving patient survival.

**Abstract:**

CCAAT/enhancer-binding protein δ (C/EBPδ) is a transcription factor involved in growth arrest and differentiation, which has consequently been suggested to harbor tumor suppressive activities. However, C/EBPδ over-expression correlates with poor prognosis in glioblastoma and promotes genomic instability in cervical cancer, hinting at an oncogenic role of C/EBPδ in these contexts. Here, we explore the role of C/EBPδ in pancreatic cancer. We determined C/EBPδ expression in biopsies from pancreatic cancer patients using public gene-expression datasets and in-house tissue microarrays. We found that C/EBPδ is highly expressed in healthy pancreatic ductal cells but lost in pancreatic ductal adenocarcinoma. Furthermore, loss of C/EBPδ correlated with increased lymph node involvement and shorter overall survival in pancreatic ductal adenocarcinoma patients. In accordance with this, in vitro experiments showed reduced clonogenic capacity and proliferation of pancreatic ductal adenocarcinoma cells following C/EBPδ re-expression, concurrent with decreased sphere formation capacity in soft agar assays. We thus report a previously unrecognized but important tumor suppressor role of C/EBPδ in pancreatic ductal adenocarcinoma. This is of particular interest since only few tumor suppressors have been identified in the context of pancreatic cancer. Moreover, our findings suggest that restoration of C/EBPδ activity could hold therapeutic value in pancreatic ductal adenocarcinoma, although the latter claim needs to be substantiated in future studies.

## 1. Introduction

Pancreatic cancer is a devastating disease with a survival outcome that is the worst of all human cancers [1]. The 5-year survival rate upon diagnosis is a little over 9% and overall mortality reaches 99% [2,3]. Due to the late onset of symptoms, only 15–20% of patients present with resectable disease, whereas the remaining patients present with metastatic or locally advanced disease, which cannot be resected. The median survival of the selected group of resectable patients, however, increases only to around 23 months whereas 5-year survival rates remain below 20% [4,5,6]. Eventually, the majority of these patients with a resectable primary tumor will succumb due to metastatic disease as well [7]. Thus, there is a clear clinical need to better understand the processes that drive pancreatic cancer and guide the development of novel avenues for rational treatment of this disease.

Ninety-five percent of pancreatic cancers arise from the exocrine compartment of the pancreas [8]. Of these exocrine tumors, pancreatic ductal adenocarcinoma comprises about 90% of all cases. In addition to pancreatic ductal adenocarcinoma, ampullary carcinoma and intrapancreatic cholangiocarcinoma may also be present within the pancreas due to their anatomical proximity, although these tumors are strictly taken no pancreatic cancers [9,10,11]. Ampullary carcinoma, with an incidence of around 0.6 cases in 100,000, arises in the ampulla of Vater, which is where the bile duct and pancreatic duct connect with the duodenum [9]. Intrapancreatic cholangiocarcinoma, with an incidence rate of 1–2 cases per 100,000, arises from epithelial cells of the bile duct (cholangiocytes) and is known as intrapancreatic bile duct cancer when it occurs where the bile duct passes through the pancreas [10,11]. The symptoms and pathology of intrapancreatic cholangiocarcinoma and pancreatic ductal adenocarcinoma are very similar and consequently these two types are difficult to distinguish.

Only a limited number of tumor suppressor genes have been formally established in pancreatic ductal adenocarcinoma. Mutations in genes such as *TP53*, *SMAD4*, *PTEN*, and *CDKN2A* are present in over 70% of pancreatic ductal adenocarcinomas, and mutations in these tumor suppressors are well known to drive tumor progression. As opposed to their clear biological relevance, mutations in tumor suppressor genes typically are of limited therapeutic value [12,13]. To improve patient treatment, the identification of tumor suppressor genes that could serve as therapeutic targets is therefore eagerly awaited.

CCAAT/enhancer-binding protein δ (C/EBPδ) is a member of the C/EBP superfamily of transcription factors, which consists of six unique members (α, β, γ, δ, ε and ζ) [14]. Soon after its discovery, C/EBPδ was implied to act as a tumor suppressor by inducing growth arrest and differentiation in breast cancer [15]. Indeed, C/EBPδ expression promotes CDC27 expression, leading to increased degradation of the cell cycle proteins cyclin D1, cyclin B1, Plk-1, and Skp2 [16]. Furthermore, expression of C/EBPδ is associated with downregulation of c-Myc and cyclin E, and upregulation of the cyclin-dependent kinase inhibitor p27 in the leukemia cell lines K562 and KCL22, leading to growth arrest and differentiation [17]. In A431 cervical cancer cells, C/EBPδ expression leads to the induction of apoptosis via the transcriptional regulation of the pro-apoptotic genes *PPARG2* and *GADD153* [18]. Moreover, C/EBPδ is involved in the regulation of pro-apoptotic gene expression and growth arrest during mammary gland involution [19,20]. In line with these data, C/EBPδ indeed acts as a tumor suppressor in breast cancer [21,22,23], ovarian serous carcinoma [24], cervical carcinoma [25], leukemia [26] and hepatocellular carcinoma [27,28].

In contrast to the presumed tumor suppressor role of C/EBPδ, several studies suggest that C/EBPδ may actually drive tumor progression in certain cancers. Indeed, C/EBPδ over-expression correlates with poor prognosis in glioblastoma [29]; it is required for efficient metastatic growth of mammary tumors [30], and drives proliferation and invasiveness of urothelial carcinoma cells, thereby driving metastatic disease leading to a reduced disease-specific survival [31]. Finally, C/EBPδ promotes tumorigenesis in the cervix by inducing aneuploidy and centromere abnormalities [32].

Taken together, the role of C/EBPδ in tumor biology seems more complex than originally anticipated and it does not seem to be a generic tumor suppressor. Instead, C/EBPδ may either suppress or promote tumor growth in a context specific manner. Here, we extend this notion by exploring the potential relevance of C/EBPδ in pancreatic cancer. We show that, despite its obvious importance for restraining cancer growth in a variety of systems, C/EBPδ takes on a tumor suppressive role in pancreatic ductal adenocarcinoma, while such effects remain insignificant, albeit not completely absent, in ampullary carcinoma or intrapancreatic cholangiocarcinoma. Moreover, we show that re-expressing C/EBPδ limits pancreatic cancer cell proliferation and future studies should elucidate whether it is of therapeutic interest in the treatment of this devastating disease.

## 2. Results

### 2.1. CEBPD mRNA Expression Is Decreased in Pancreatic Ductal Adenocarcinoma Tissue

**Figure 1:**
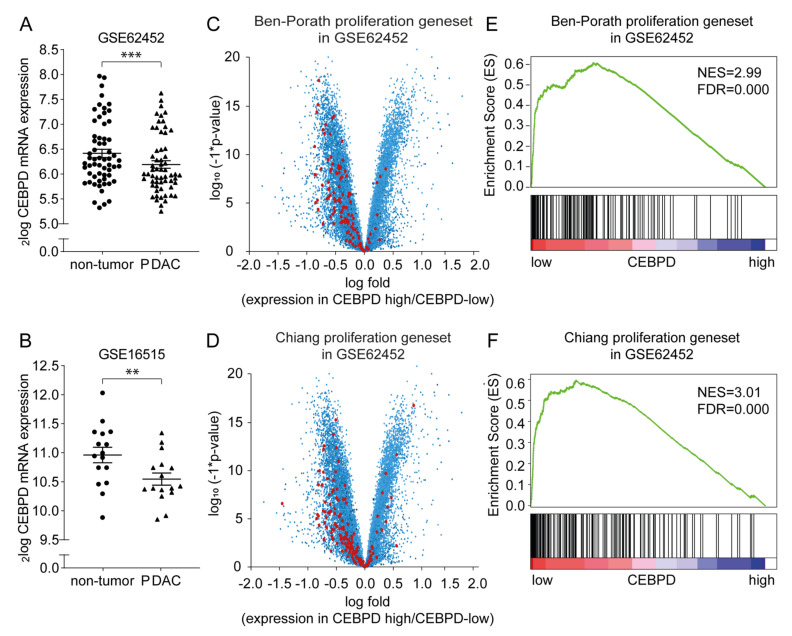
Analysis of publicly available datasets reveals C/EBPδ as a potential tumor suppressor in pancreatic ductal adenocarcinoma. (**A**,**B**) *CEBPD* gene expression in pancreatic ductal adenocarcinoma biopsies and adjacent healthy pancreatic tissue biopsies derived from GSE62452 (**A**) and GSE16515 (**B**). ** *p* < 0.01, *** *p* < 0.001 compared to healthy control tissue. Lines indicate the mean ± SEM. (**C**,**D**) Volcano plot of statistical significance against fold change between high and low *CEBPD* samples. Each data point represents a gene, while genes from the Ben-Porath [35] (**C**) or Chiang [36] (**D**) proliferation gene signatures are highlighted in red. Negative fold changes imply higher expression of a gene in the *CEBPD*-low group and vice versa. (**E**,**F**) Gene set enrichment analysis (GSEA) using the Ben-Porath (**E**) or Chiang (**F**) proliferation gene signatures on patients with high or low *CEBPD* expression levels in GSE62452. GSEA: gene set enrichment analysis, NES: normalized enrichment score, FDR: false discovery rate.

To assess whether C/EBPδ may act as a tumor suppressor in pancreatic cancer, we first analyzed *CEBPD* mRNA expression levels in pancreatic ductal adenocarcinomas and in adjacent control tissue in publicly available gene expression datasets (GSE62452 [33] and GSE16515 [34]). As shown in Figure 1, *CEBPD* mRNA expression was observed in both tumor and non-tumor tissue in both datasets analyzed. Interestingly, however, *CEBPD* expression was decreased in tumor tissue as compared to the control tissue in both the GSE62452 (Figure 1A) and GSE16515 (Figure 1B) dataset. Subsequently, patients of the datasets were dichtomerized into *CEBPD*-high and *CEBPD*-low groups whereupon differential gene expression analysis between these groups revealed enhanced expression of genes from published proliferation signatures [35,36] in patients of the *CEBPD*-low group opposed to the *CEBPD*-high group (data for GSE62452 shown in Figure 1C,D and Table S1). To corroborate these findings and provide statistics, we next performed gene set enrichment analyses of the proliferation signatures on the pancreatic cancer datasets. Samples were dichotomized by median CEBPD expression, and analysis showed a significant association with both signatures in the datasets (Figure 1E for the Ben-Porath proliferation gene set [35] and Figure 1F for the Chiang gene set [36], respectively, in GSE62452).

### 2.2. C/EBPδ Protein Levels Are Decreased in Pancreatic Ductal Adenocarcinoma but Not in Ampullary Carcinoma or Intrapancreatic Cholangiocarcinoma

**Figure 2:**
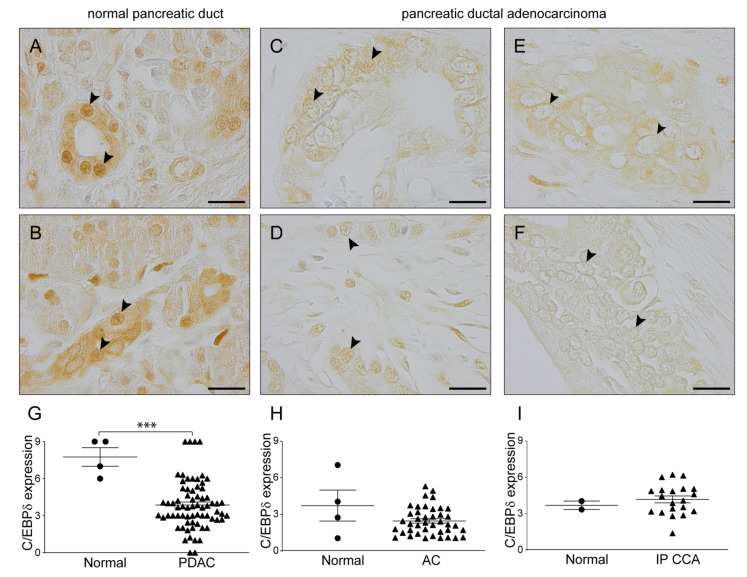
C/EBPδ expression is decreased in the nuclei of pancreatic ductal adenocarcinoma cells. Immunohistochemical staining of C/EBPδ shows strong expression in healthy pancreatic tissue (**A**,**B**) and decreased (**C**,**D**) or no expression (**E**,**F**) in pancreatic ductal adenocarcinoma. Representative tumor nuclei are indicated with black arrowheads. Quantification of nuclear C/EBPδ expression levels in healthy control tissue (**G**–**I**), pancreatic ductal adenocarcinoma (**G**), ampullary carcinoma (**H**) and intrapancreatic cholangiocarcinoma (**I**). *** *p* < 0.001 compared to healthy control tissue. Lines indicate the mean ± SEM. PDAC: pancreatic ductal adenocarcinoma, AC: ampullary carcinoma, IP CCA: intrapancreatic cholangiocarcinoma. Scale bars: 20 µm.

C/EBPδ is a ubiquitously expressed transcription factor which is not specific to epithelial (tumor) cells but also expressed in cell types of the stromal compartment including pancreatic stellate cells [37]. Indeed, *CEBPD* expression levels correlate with stromal gene signatures in five out of six publicly available datasets containing pancreatic ductal adenocarcinoma (PDAC) samples [34,38,39,40,41,42] as determined using ESTIMATE [43] (Table S2 and Figure S1). Moreover, for transcriptional activity of C/EBPδ, only nuclear expression is relevant and gene expression levels of tissue biopsies such as those used in the bioinformatics experiments described above may consequently not accurately reflect the C/EBPδ activity that is relevant for tumor cell biology. To circumvent these confounding effects of whole biopsy gene expression analysis, we subsequently immunohistochemically analyzed nuclear C/EBPδ protein levels in tumor cells of a cohort of 67 pancreatic ductal adenocarcinoma patients using normal duct epithelium as control. C/EBPδ was highly expressed in normal, non-tumorigenic, pancreatic ductal cells (Figure 2A,B). The vast majority of normal pancreatic ductal cells showed strong nuclear C/EBPδ staining. Interestingly, C/EBPδ expression was significantly decreased in pancreatic ductal adenocarcinoma cells (Figure 2C–G). Although some cytoplasmic staining was still observed in pancreatic ductal adenocarcinoma biopsies, most of the tumor cell nuclei were negative for C/EBPδ or the intensity was very much decreased compared to normal tissue.

To determine whether the observed decrease in nuclear C/EBPδ expression in pancreatic cancer is specific to pancreatic ductal adenocarcinomas or whether it is a more general phenomenon, we also determined nuclear C/EBPδ expression levels in ampullary carcinoma and intrapancreatic cholangiocarcinoma. We found that C/EBPδ expression levels were relatively low in the nuclei of normal intestinal epithelial cells within the ampulla of Vater (Figure 2H) and in normal cholangiocytes (Figure 2I) compared to normal pancreatic ductal cells. Moreover, nuclear C/EBPδ expression was not further decreased in tumor cells of these cancer types and semi-quantitative analysis of C/EBPδ expression levels showed no significant difference between normal and tumor cell nuclei in these patients (Figure 2H,I).

### 2.3. C/EBPδ Protein Expression Is Associated with Regional Lymph Node Involvement and Correlates with Overall Survival in Pancreatic Ductal Adenocarcinoma

**Figure 3:**
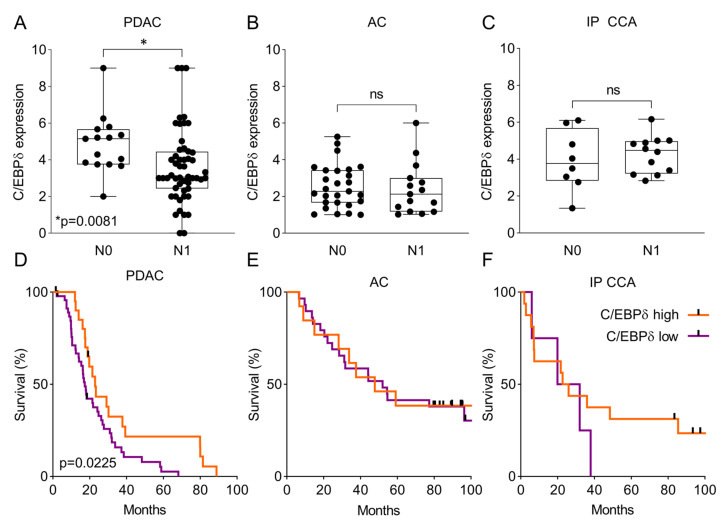
C/EBPδ expression correlates with regional lymph node metastasis and overall survival in pancreatic ductal adenocarcinoma patients. (**A**–**C**) C/EBPδ expression levels in patients with and without regional lymph node metastasis (N0: negative regional lymph nodes, N1: positive regional lymph nodes) in pancreatic ductal adenocarcinoma (**A**), ampullary carcinoma (**B**) and intrapancreatic cholangiocarcinoma (**C**). (**D**–**F**) Overall survival in pancreatic ductal adenocarcinoma (**D**), ampullary carcinoma (**E**) and intrapancreatic cholangiocarcinoma (**F**) in different groups of C/EBPδ expression levels.Vertical black bars in Kaplan-Meier plots represent censored patients. ns: not significant.

To assess the potential clinical relevance of reduced C/EBPδ expression in pancreatic ductal adenocarcinoma, we first determined whether C/EBPδ correlated with lymph node involvement in these patients. As shown in Figure 3A and Table 1, C/EBPδ expression in the nuclei of primary tumors was significantly decreased in patients with tumor cell positive regional lymph nodes (N1) compared to patients without tumor cell positive regional lymph nodes (N0). This association of C/EBPδ protein levels with N-status was absent in ampullary carcinoma (Figure 3B) and intrapancreatic cholangiocarcinoma (Figure 3C). In line with metastasis to local lymph nodes, decreased nuclear C/EBPδ expression was also significantly correlated with shorter overall survival in pancreatic ductal adenocarcinoma patients (Figure 3D). Median survival of pancreatic ductal adenocarcinoma patients in the lower half of C/EBPδ expression was 16.9 months, whereas median survival of patients in the upper half of C/EBPδ expression was 22.2 months (*p* < 0.05). Compared to pancreatic ductal adenocarcinoma, patients with ampullary carcinoma (Figure 3E) or intrapancreatic cholangiocarcinoma (Figure 3F) showed a longer median overall survival of 49.9 and 24.4 months, respectively (versus 18.3 months for the pancreatic ductal adenocarcinoma patients). As expected based on similar C/EBPδ expression and the lack of association with regional lymph node involvement, C/EBPδ expression also did not correlate significantly with overall survival in ampullary carcinoma and intrapancreatic cholangiocarcinoma. These results lead to the notion that C/EBPδ may act as a tumor suppressor in pancreatic ductal adenocarcinoma, while this relation is not observed in ampullary carcinoma or intrapancreatic cholangiocarcinoma.

### 2.4. C/EBPδ Modulates Oncogenesis of Pancreatic Ductal Adenocarcinoma Cells

#### 2.4.1. C/EBPδ Over-Expression Reduces Proliferation of Pancreatic Ductal Adenocarcinoma Cells

**Figure 4:**
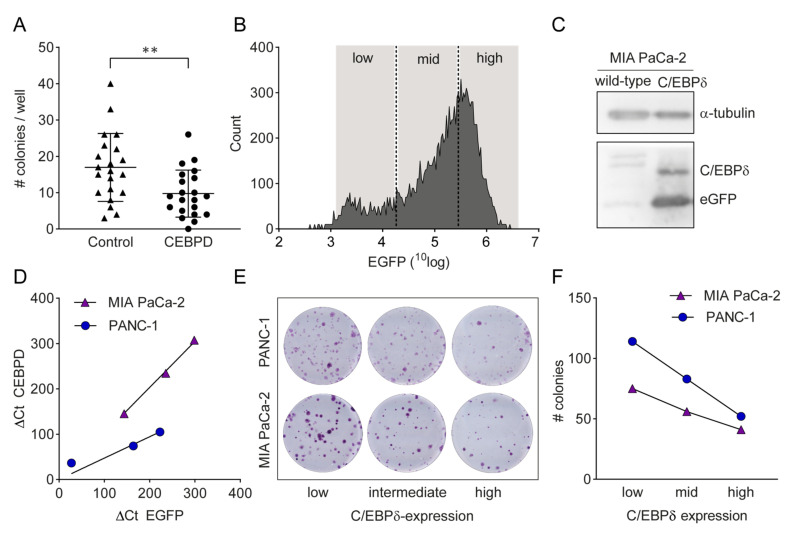
C/EBPδ over-expression inhibits clonogenicity in PANC-1 and MIA PaCa-2 cells. (**A**) Average number of colonies grown out from 100 C/EBPδ-over-expressing or control-transfected PANC-1 cells per well in three independent experiments (*N* = 21). Lines indicate the mean ± SEM. ** *p* < 0.005. Out of 2100 control-transfected cells, 356 grew into a colony. Only 205 out of 2100 C/EBPδ over-expressing cells grew out a colony. (**B**) Histogram and gating of *CEBPD-*IRES*-EGFP*-transduced MIA PaCa-2 cells. (**C**) Western blot showing over-expression of C/EBPδ and eGFP protein after transfection. The uncropped blots are provided in Figure S2. (**D**) *CEBPD* and *EGFP* mRNA expression of *CEBPD*-IRES-*EGFP*-transduced cells sorted by EGFP-fluorescence. Quantitative RT-PCR confirmed elevated *CEBPD* mRNA expression in cells expressing high *EGFP* mRNA (MIA PaCa-2: R^2^ = 0.9975, * *p* < 0.05; PANC-1: R^2^ = 0.9724, *p* = 0.0531). (**E**) Clonogenic assay of PANC-1 and MIA PaCa-2 cells expressing C/EBPδ at varying levels. (**F**) C/EBPδ expression correlates with colony formation efficiency. Plotting the number of colonies from Figure 4E against the respective *CEBPD* mRNA expression shows a dependency of colony formation efficiency on *CEBPD* levels in PANC-1 and MIA PaCa-2 cells.

To further validate the potential tumor-suppressive effects of C/EBPδ in pancreatic ductal adenocarcinoma, we conducted in vitro clonogenic assays with two commonly used pancreatic ductal adenocarcinoma cell lines, PANC-1 and MIA PaCa-2. From publicly available mRNA expression datasets, we know that both these cell lines show low baseline expression levels of *CEBPD* which is in line with the low expression levels observed in pancreatic ducal adenocarcinoma patients, making them a solid model for C/EBPδ over-expression studies. First, we assessed the effect of transient C/EBPδ over-expression on the clonogenic capacity of PANC-1 cells. To this end, 100 control- or *CEBPD*-transfected cells were seeded into 24-well plates after which colony formation was observed over time. As shown in Figure 4A, we observed a significant reduction in clonal outgrowth by C/EBPδ over-expressing cells as compared to control cells (*p* < 0.005). To corroborate these findings, we next seeded single control- or *CEBPD*-transfected cells in 96-well plates. Similar to the 100 cells per well approach, *CEBPD* over-expression decreased the clonogenic capacity of PANC-1 cells. Indeed, *CEBPD*-over-expressing cells showed a clonogenic capacity of 8% (*N* = 23 colonies out of 288 single cells) as compared to 16% in control-transfected cells (*N* = 46 colonies out of 288 single cells), constituting a decrease of 50% in clonogenic capacity (χ^2^ test, *p* = 0.0032).

As C/EBPδ is expected to dilute out in transient transfection experiments, we next performed stable transfection experiments. To this end, PANC-1 and MIA PaCa-2 cells were transduced with a *CEBPD*-IRES-*EGFP* over-expression plasmid; after which, cells were sorted into three fractions based on eGFP fluorescence (Figure 4B). Importantly, both C/EBPδ and eGFP were indeed over-expressed in transduced cells (Figure 4C) and *CEBPD* expression correlated with *EGFP* expression (Figure 4D). Subsequent clonogenic experiments with 500 transduced cells of each fraction seeded in 12-well plates confirmed the transient transfection experiments by showing that C/EBPδ expression was negatively correlated with colony formation in both cell lines (PANC-1 low expression: 114 colonies; intermediate expression: 83 colonies; high expression: 52 colonies. MIA PaCa-2 low expression: 75 colonies; intermediate expression: 56 colonies; high expression: 41 colonies) (Figure 4E,F). Altogether, these in vitro experiments imply that C/EBPδ indeed limits the growth of pancreatic ductal adenocarcinoma cells.

#### 2.4.2. A Tet-on System Reveals Dose-Dependent Effects of C/EBPδ on Proliferation and Clonogenicity

**Figure 5:**
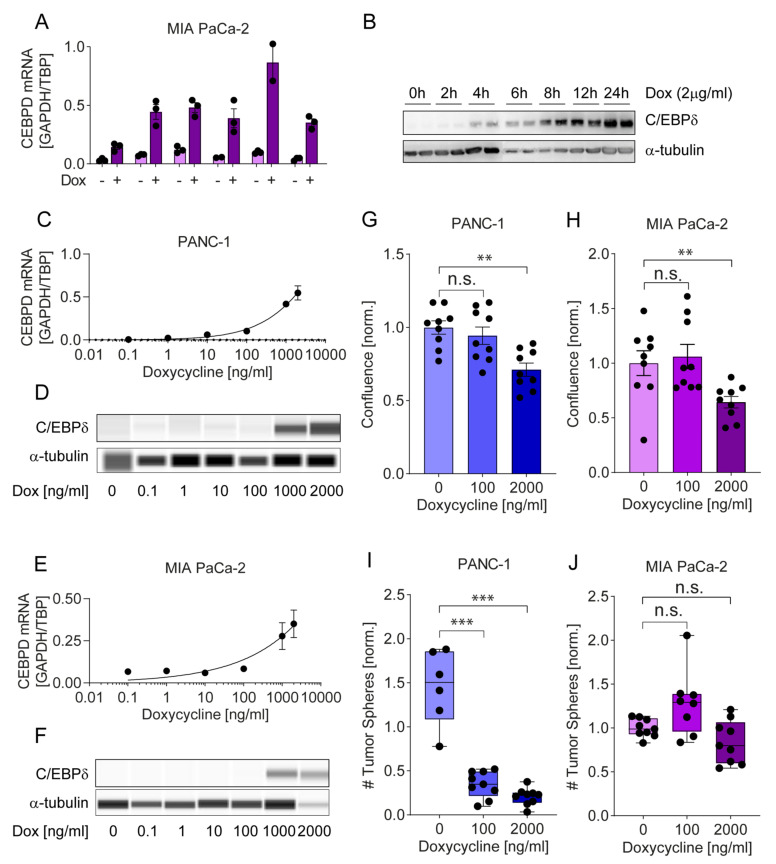
Doxycycline dose-dependent induction of C/EBPδ regulates proliferation and tumor sphere formation. (**A**) Single clones of MIA PaCa-2 cells, transduced with a doxycycline-inducible C/EBPδ over-expression plasmid, show increased C/EBPδ mRNA levels upon induction. (**B**) C/EBPδ induction in MIA PaCa-2 cells over time. The uncropped blots are provided in Figure S3A (**C**–**F**) Doxycycline dose-dependently induces C/EBPδ in both transduced MIA PaCa-2 and PANC-1 cells. The uncropped blots are provided in Figure S3B,C. (**G**,**H**) Proliferation is mildly decreased by 100 ng/mL and significantly reduced by 2000 ng/mL doxycycline in PANC-1 (** *p* = 0.0012) and MIA PaCa-2 (** *p* = 0.0078) cells. (**I**,**J**) Tumor sphere formation in soft agar is significantly reduced by 100 and 2000 ng/mL doxycycline in PANC-2 (*** *p* = 0.0004). A similar yet not significant trend is observed in MIA PaCa-2 with 2000 ng/mL doxycycline. Dox: Doxycycline. n.s.: not significant, *** *p* < 0.001.

To widen our understanding of the effects of C/EBPδ on pancreatic adenocarcinoma cells, we turned to a doxycycline-inducible Tet-On system. As shown in Figure 5A–F, this system permits tightly controlled expression of C/EBPδ with induction levels at the 2000 ng/mL doxycycline dose, mimicking the fold-change in protein expression observed in tumor versus normal patient samples. As observed with constitutive over-expression, controlled over-expression of C/EBPδ curbed proliferation of pancreatic adenocarcinoma cells. Interestingly, subjecting the cells to a concentration of 100 ng/mL doxycycline, which minimally induces C/EBPδ, already moderately reduced proliferation of PANC-1 while a concentration of 2000 ng/mL significantly reduced proliferation in both PANC-1 and MIA PaCa-2 (Figure 5G,H).

Next, we assessed the limiting effects of C/EBPδ on colony formation of pancreatic adenocarcinoma cells. Seeding single cells in 96-well plates, we found that 2000 ng/mL doxycycline reduced single cell outgrowth by 32% in MIA PaCa-2 and by 12**%** in PANC-1cells (data not shown). The observed reduction in clonogenicity was lower compared to that seen in the constitutive system shown in Figure 4 which is well in accordance with the difference in C/EBPδ expression between the two systems and again emphasizes that these effects are dose-dependent on C/EBPδ.

In addition to two-dimensional clonogenic and proliferation assays, we next assessed anchorage-independent growth in three-dimensional soft agar sphere formation assays. This assay constitutes a well-recognized measure of stemness and malignant potential owed to anchorage-independent out-growth of a single cell [44]. As in two-dimensional clonogenic assays, we also observed a decrease in tumor sphere formation when comparing doxycycline-treated C/EBPδ over-expressing cells to untreated low C/EBPδ expressing cells. Interestingly, 100 ng/mL doxycycline already significantly limited sphere formation in PANC-1 cells but had no effect in MIA PaCa-2 cells. The 2000 ng/mL doxycycline dose again drastically suppressed sphere formation by PANC-1 and also, to some extent, reduced the number of spheres formed by MIA PaCa-2 cells although this difference did not reach significance (Figure 5I,J).

#### 2.4.3. Silencing C/EBPδ Enhances Proliferation of Pancreatic Ductal Adenocarcinoma Cells

**Figure 6:**
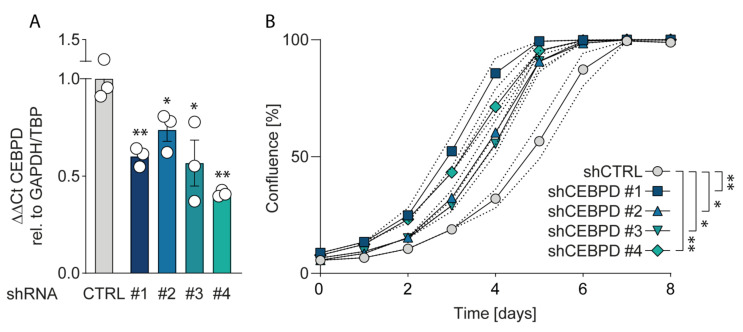
Silencing C/EBPδ enhances proliferation of Capan-2 cells. (**A**) Quantitative RT-PCR shows decreased levels of *CEBPD* mRNA in Capan-2 cells stably expressing shRNAs against *CEBPD.* (*p* = **0.0058, *0.0429, *0.0338 and **0.001 for shCTRL vs. shCEBPD #1, #2, #2 and #4, respectively). (**B**) Proliferation of *CEBPD*-silenced Capan-2 cells is significantly increased compared to control cells. (*p* = **0.0013, *0.011, *0.0146 and **0.0013 for shCTRL vs. shCEBPD #1, #2, #2 and #4, respectively).

Considering the finding that over-expressing C/EBPδ reduces the proliferation of pancreatic ductal adenocarcinoma cells, thereby decreasing their malignant potential, we next asked whether silencing C/EBPδ would conversely trigger proliferation. As both PANC-1 and Mia PaCa-2 cells already express very low or undetectable C/EBPδ levels, we turned to Capan-2 cells that, according to public gene expression datasets, show the highest *CEBPD* levels of all routinely used pancreatic cancer cell lines (Figure S4) [45,46]. Interestingly, shRNA *CEPBD*-silenced Capan-2 cells (silencing efficiency in Figure 6A) indeed showed increased proliferation rates as compared to control-silenced cells (Figure 6B). Notably, more efficient silencing corresponded to progressively increased proliferative capabilities, underscoring the dose-dependent effect of C/EBPδ on pancreatic adenocarcinoma cell behavior.

## 3. Discussion

C/EBPδ has been found to negatively affect tumor growth by inducing growth arrest and differentiation [21,22,23,24,25,26,27,28]. Recently, however, C/EBPδ was also suggested to drive tumor progression and/or metastasis in certain tumor types [29,30,31,32], suggesting that C/EBPδ may either suppress or promote tumor progression in a context specific manner. Here, we explore the importance of C/EBPδ in pancreatic cancer and show that C/EBPδ harbors tumor suppressor activity in pancreatic ductal adenocarcinoma and that re-expressing C/EBPδ in pancreatic adenocarcinoma cells curbs clonogenicity and proliferation.

Data mining of publicly available microarray datasets showed that *CEBPD* gene expression is significantly decreased in pancreatic ductal adenocarcinomas versus healthy pancreatic tissue. Although of obvious interest, whole tumor biopsy *CEBPD* expression levels do not necessarily correspond to C/EBPδ activity in tumor cells. In addition to confounding expression of *CEBPD* in stromal cells, expression levels in whole tumor biopsies may also not fully represent nuclear C/EBPδ activity. Subsequent immunohistochemical analyses of tissue arrays underscore this notion and indeed show both stromal and cytoplasmic staining. Of interest, in pancreatic tissue biopsies, healthy ductal cells express high nuclear levels of C/EBPδ which is at odds with the general notion that C/EBPδ expression is typically low under normal conditions [47]. More importantly, C/EBPδ levels are dramatically reduced in ductal adenocarcinoma cells as compared to normal ductal cells. As C/EBPδ induces growth arrest [16,17], it is tempting to speculate that the loss of C/EBPδ conversely facilitates tumorigenesis. Such a role of C/EBPδ would be in line with previous studies showing that C/EBPδ is a tumor suppressor in leukemia, breast cancer, hepatocellular carcinoma and cervical cancer [21,22,23,24,25,26,27,28].

We were able to confirm that C/EBPδ expression is low in MIA PaCa-2 and PANC-1, two commonly used pancreatic ductal adenocarcinoma cell lines. This is in line with our observations in pancreatic cancer patients and with a potential role of C/EBPδ as a tumor suppressor in pancreatic cancer. More importantly, their low C/EBPδ expression makes these cells suitable model systems for C/EBPδ re-expression studies with the assumption that rescuing C/EBPδ expression would reduce their tumorigenic capacity in case C/EBPδ acts as a genuine tumor suppressor. PANC-1 and MIA PaCa-2 cells indeed showed decreased proliferation and clonogenicity upon C/EBPδ re-expression. Additionally, these effects were dose dependent, implying a relation between tumorigenicity and C/EBPδ expression levels. Conversely, shRNA-dependent silencing of C/EBPδ enhances proliferation in a dose-dependent manner strongly suggesting that C/EBPδ expression levels negatively correlate with the proliferative capacity of pancreatic ductal adenocarcinoma cells.

Next to driving proliferation and clonogenicity of pancreatic ductal adenocarcinoma cells, loss of C/EBPδ also seems to promote anchorage-independent growth. This clonogenic capacity in a three-dimensional, anchorage-free environment is considered a key hallmark of oncogenic transformation and is considered the most accurate and stringent in vitro assay for detecting malignant transformation of cells. We found a marked reduction in the number of spheres formed by two pancreatic adenocarcinoma cell lines upon re-expression of C/EBPδ. Interestingly, although C/EBPδ re-expression limits sphere formation in both PANC-1 and MIA PaCa-2 cells, PANC-1 cells appear to be more susceptible to reversing oncogenic properties upon C/EBPδ induction. Although the precise mechanism underlying the reduction in anchorage-independent growth of pancreatic adenocarcinoma cancer cell remains to be established, it is tempting to speculate that these data explain the correlation found between C/EBPδ expression and lymph node invasion observed in our patient cohort. Indeed, anchorage-independent growth is strongly associated with the metastatic potential of cancer cells [44]. Irrespective of the actual mechanism, the reduced overall survival in pancreatic ductal adenocarcinoma patients with low C/EBPδ levels might be directly linked to the increased lymph node metastases in these patients.

Taken together, our results point towards a clear direction where C/EBPδ regulates the proliferation and clonogenic capacities of PDAC cells. However, as two-dimensional monoculture experiments in vitro cannot account for important factors, such as stromal and immune infiltration, in vivo validation of these findings is urgently needed to manifest the notion that C/EBPδ might act as a tumor suppressor in PDAC.

C/EBPδ is obviously not the first tumor suppressor identified in pancreatic adenocarcinoma. Indeed, genes such as *TP53*, *SMAD4*, *PTEN*, and *CDKN2A* are well-known tumor suppressors and mutations in these genes, which are present in over 70% of pancreatic ductal adenocarcinomas, drive tumor progression. As opposed to the other classical tumor suppressors, C/EBPδ seems, however, neither hypermethylated, nor mutated, lost or deleted in pancreatic adenocarcinoma (re-analysis of previously published data [48,49,50]) suggesting re-activation of C/EBPδ may hold therapeutic promise in the setting of pancreatic adenocarcinoma. Although C/EBPδ re-expression indeed limits proliferation and clonogenicity in pancreatic adenocarcinoma cell lines, future studies should prove or refute this hypothesis.

The mechanism via which C/EBPδ exerts its tumor suppressive and anti-metastatic effects remains elusive. In an attempt to uncover the underlying mechanism, we have investigated the expression of different putative targets of C/EBPδ involved in cell cycle progression, apoptosis, stemness and the leading-edge genes of the above described gene set enrichment analyses (GSEAs) (Figure S5). Enhanced expression of CDKN1A (p21) along with suppressed cyclin-dependent kinases CDK1, CDK2 and CDK6 point towards cell cycle arrest as a main mechanism of the observed C/EBPδ-induced effects in PDAC cells. Interestingly, however, C/EBPδ also appears to affect almost all of the other investigated pathways to some degree. Hence, the data are not as straightforward as expected and do not allow firm conclusions on the downstream pathways affected by C/EBPδ. High-throughput RNAseq experiments will be deployed to uncover the major driving mechanism underlying C/EBPδ’s role in PDAC. These will be valuable in the view of molecular biology as well as for the discovery of new clinical targets in the treatment of PDAC.

To date, several agents have been described to effectively induce C/EBPδ expression. Among these activators are interleukin-6, which elicited growth-inhibiting effects on LNCaP prostate cancer cells via C/EBPδ activation [51], 1-(2-hydroxy-5-methylphenyl)-3-phenyl-1, 3-propanedione (HMDB), which attenuated the growth of A431 epidermoid carcinoma xenografts in severe combined immunodeficient mice [18,27], and metformin, which induced autophagy of Huh7 liver cancer cells via C/EBPδ activation [52]. Next to that, C/EBPδ has been induced by various external stimuli in the inflammatory context [53]. Preliminary experiments did not show a significant effect of any of these compounds on *CEBPD* expression in pancreatic adenocarcinoma cells and further studies are needed to find potent upstream regulators of C/EBP δ in this context.

In contrast to our observation that the loss of C/EBPδ is associated with increased lymph node metastasis and subsequent poor prognosis, it has recently been shown that C/EBPδ amplification drives tumor metastasis in urothelial carcinoma [31]. These latter data are in line with a study showing that C/EBPδ over-expression correlates with poor prognosis in glioblastoma [29]. A picture thus emerges that C/EBPδ may act either as a tumor suppressor or as an oncogene in a context-dependent manner. In line with this notion, we show here that C/EBPδ levels in two other tumors that are located within the pancreas, i.e., ampullary carcinoma and intrapancreatic cholangiocarcinoma, do not differ significantly from control levels in normal intestinal epithelial cells and cholangiocytes, respectively. More importantly, we could not identify a correlation of either patient survival or lymph node status with C/EBPδ expression in ampullary carcinoma or intrapancreatic cholangiocarcinoma patients based on which we propose that C/EBPδ is unlikely to act as a tumor suppressor in these cancers. Although it may be tempting to suggest that the baseline expression of C/EBPδ in healthy tissues can generally determine the sensitivity of an arising tumor to the tumor suppressive effects of C/EBPδ, the questions of what determines whether C/EBPδ acts as tumor promotor or tumor suppressor remains to be answered.

## 4. Materials and Methods

### 4.1. Mining of Publicly Available RNA Microarray Datasets

Datasets were derived from Gene Expression Omnibus [48] using the R2 microarray analysis and visualization platform [46]. *CEBPD* expression levels were derived from two different datasets, i.e., GSE62452 (updated version of GSE28735 [33] with an extra 16 tumor and control biopsies) and GSE16515 [34]. From the GSE16515 dataset, we only included patients of which paired tumor and adjacent non-tumor biopsies were available. Gene set enrichment analysis (GSEA) was performed using the Broad Institute tool [49]. Samples were dichotomized by median CEBPD expression. *p*-values indicating the significance of enrichment were determined by 1000 permutations using the Ben-Porath [35] and Chiang [36] proliferation gene sets.

### 4.2. Tissue Microarray (TMA)

Formalin-fixed, paraffin-embedded biopsies from 129 pancreatic cancer cases between the years of 1983 and 2015 were used for compilation of tissue microarrays (TMAs) using routine procedures. Biopsies were selected from the archives of the Pathology Department at the Amsterdam University Medical Center, Amsterdam. The study was approved by the investigator’s institutional review boards. Patients with a known previous malignancy in another organ were excluded from the analysis. The current study included 89 men (69.8%) and 40 women (30.2%), their ages range from 47 to 83 years, with a mean (±SD) of 64.4 (±8.9) and median of 65 years (Table 2). Sixty-seven patients were diagnosed with pancreatic ductal adenocarcinoma, 42 with ampullary carcinoma and 20 with intrapancreatic cholangiocarcinoma. For 104 of the patients, 3 cores were available each (43 pancreatic ductal adenocarcinoma, 20 intrapancreatic cholangiocarcinoma, 41 ampullary carcinoma). Each core received an individual score as described in 4.4. For 25 patients, only one core was available (24 pancreatic ductal adenocarcinoma, 1 ampullary carcinoma). From each of these cores, three tumor-cell containing locations were selected at random and nuclear C/EBPδ staining was quantified as described in 4.4. Six biopsies of normal pancreas tissue were included to serve as healthy control and C/EBPδ staining was quantified as described in 4.4.

### 4.3. Immunohistochemistry

C/EBPδ stainings were performed essentially as described before [54,55,56,57] with minor modifications. Four micron-thick paraffin embedded tissue sections were deparaffinized and treated with 0.3% H_2_O_2_ in methanol for 15 min to block endogenous peroxidase activity. Subsequently, slides were blocked with Ultra V block (#TA-125-UB; Thermo Fisher Scientific, Waltham, MA, USA) and incubated with a rabbit polyclonal antibody against C/EBPδ (#GWB-MM818H; GenWay Biotech, San Diego, CA, USA) in a 1:1000 dilution in PBS at 4 °C overnight. The next day, slides were incubated with Powervision poly-HRP anti rabbit IgG (#DPVM-55HRP; Immunologic, Duiven, Netherlands) for 30 min at room temperature and stained using 3,3’Diaminobenzidine (Bright DAB, #BS04-999; Immunologic). Hematoxylin (1:10 in demineralized H2O) was applied as counterstaining.

### 4.4. Quantification of C/EBPδ Protein Levels

C/EBPδ-stained slides were reviewed by three independent pathologists in a blinded fashion to the clinical status of the patients. Specimens with conflicting scores were re-evaluated until consensus was reached. Nuclear C/EBPδ expression in tumor cells was scored based on the percentage of positively stained nuclei and the intensity of the positive staining. The percentage of positive nuclei for each core was scored on a scale from 0 to 3 (0: no positive nuclei, 1: less than 30% positive nuclei, 2: between 30–70% positive nuclei, 3: more than 70% positive nuclei) whereas the intensity of the positive nuclei was scored on a scale from 1 to 3 (1: low intensity, 2: intermediate intensity, 3: high intensity). The intensity score (i.e., the average score of 10 randomly selected cells in three different areas of each core) was finally multiplied by the percentage score leading to a theoretical maximum score of 9. For survival analysis Kaplan–Meier curves were constructed using GraphPad Prism 6.0 (GraphPad Software Inc., La Jolla, CA, USA) whereby C/EBPδ expression levels were divided into an upper and a lower half based on the range observed in each type of tumor.

### 4.5. Cell Lines and Cell Culture Reagents

Human PANC-1, MIA PaCa-2 and CAPAN-2 pancreatic cancer cell lines (ATCC, Manassas, VA, USA) were cultured in high glucose in Dulbecco’s Modified Eagle Medium (Gibco, Thermo Fischer Scientific, Waltham, MA, USA) supplemented with 10% (*v/v*) fetal calf serum (FCS; Serana, Pessin, Germany), 2% (*v/v*) penicillin–streptomycin (Gibco, Thermo Fischer Scientific, Waltham, MA, USA) and 2 mM L-glutamine (Lonza, Basel, Switzerland). Cells were incubated in 5% CO_2_ incubators at 37 °C. All cell lines were tested mycoplasma-negative and their identities have been confirmed by STR-profiling.

### 4.6. RNA Isolation, cDNA Synthesis and RT-qPCR

To determine mRNA expression levels, RNA was extracted using the NucleoSpin^®^ RNA-extraction Kit (Macherey-Nagel GmbH and Co. KG, Düren, Germany), according to the supplier’s protocol for cultured cells. Eluted RNA was analyzed spectrophotometrically using the NanoDrop 2000. All samples were treated with RQ1 RNAse-Free DNAse (Promega Benelux BV, Leiden, Netherlands) and reverse-transcribed into cDNA using M-MLV Reverse Transcriptase (Promega Benelux BV, Leiden, Netherlands), random hexamers (Fisher scientific, Landsmeer, Netherlands) and 10 mM dNTPs (Fermentas, Fisher scientific, Landsmeer, Netherlands). The SensiFAST™ SYBR^®^ No-ROX Kit (GC biotech, Waddinxveen, Netherlands) was used to perform real-time quantitative RT-PCR on a LightCycler^®^ 480 Instrument II (Roche Molecular Systems, Inc., Almere, Netherlands). *CEBPD* expression levels were normalized to the expression of the reference genes *TBP* and *GAPDH* or *TBP (EGFP* vs. *CEBPD)* using the following primers.

*GAPDH* forward primer (5′-3′): AAGGTGAAGGTCGGAGTCAAC;

*GAPDH* reverse primer (5′-3′): TGGAAGATGGTGATGGGATT;

*TBP* forward primer (5′-3′): ATCCCAAGCGGTTTGCTGC;

*TBP* reverse primer (5′-3′): ACTGTTCTTCACTCTTGGCTC;

*CEBPD* forward primer (5′-3′): GCAGAAGTTGGTGGAGCTGT; 

*CEBPD* reverse primer (5′-3′): TTACCGGCAGTCTGCTGTC;

*EGFP* forward primer (5′-3′): AGCTGACCCTGAAGTTCATCTG;

*EGFP* reverse primer (5′-3′): AAGTCGTGCTGCTTCATGTG;

CDK1 forward primer (5′-3′): CCCTTTAGCGCGGATCTA;

CDK1 reverse primer (5′-3′): ATGGCTACCACTTGACCTGT;

CDK2 forward primer (5′-3′): GAAAAGATCGGAGAGGGCA;

CDK2 reverse primer (5′-3′): ACCCTCAGTCTCAGTGTCCA;

CDK4 forward primer (5′-3′): TCTATGGTCGGGCCCTCTG;

CDK4 reverse primer (5′-3′): TCAGATCAAGGGAGACCCT;

CDK6 forward primer (5′-3′): CTGCAGGGAAAGAAAAGTGC;

CDK6 reverse primer (5′-3′): TTCCCTCCTCGAAGCGAAG;

CDKN1A forward primer (5′-3′): GCATGATCTGAGTTAGGTCAC;

CDKN1A forward primer (5′-3′): GACATGGCGCCTGAACAGA;

BCL-2 forward primer (5′-3′): GGTGGGGTCATGTGTGTGG;

BCL-2 reverse primer (5′-3′): CGGTTCAGGTACTCAGTCATCC;

BCL-XL forward primer (5′-3′): AGAGAACAGGACTGAGGCCC;

BCL-XL reverse primer (5′-3′): TCAAAGCTCTGATATGCTGTCCC;

CD44 forward primer (5′-3′): AAGGTGGAGCAAACACAACC;

CD44 reverse primer (5′-3′): CTGAGACTTGCTGGCCTCTC;

TOP2A forward primer (5′-3′): TACATCCAAGGGTGGCAGAC;

TOP2A reverse primer (5′-3′): CCTGATGTGCTTTTACTGCAACA;

TTK forward primer (5′-3′): CATCAACATGGCATTGTTCAC;

TTK reverse primer (5′-3′): TCTGGTTGCATTTGGTTTGC;

ASPM forward primer (5′-3′): GTTGCAGACAAAGGCGGAAG;

ASPM reverse primer (5′-3′): CCTACTTCGTACATCAGAGGCTC;

ANLN forward primer (5′-3′): TTCCCAAAGGGATGGCGATG;

ANLN reverse primer (5′-3′): GGAGAAGTAGCTTTCACAGAGC;

### 4.7. Gene Transfection and Transduction

PANC-1 cells were transfected with *pHEF-1TIG-CEBPD-IRES-EGFP* or *pHEF-1TIG-IRES-EGFP* using the Biontex K2 transfection system (Biontex, München, Germany) according to the supplier’s protocol. For constitutive over-expression of C/EBPδ, a third-generation lentiviral system using *pHEF-1TIG-CEBPD-IRES-EGFP* or *pHEF-1TIG-IRES-EGFP*, *pMDLg/pRRE* (Addgene #12251), *pRSV-Rev* (Addgene # 12253) and *pMD2.G* (Addgene #12259) was employed to stably transduce PANC-1 and MIA-PaCa-2 cells. We have found that lentivirus production was enhanced in HEK293T cells expressing an shRNA targeting *CEBPD* (Figure S6B). Therefore, we used HEK293T cells stably transduced with an shRNA targeting *CEBPD* (MERCK MISSION^®^ TRC-No, TRCN0000013969, clone ID NM_005195.2-271s1c1) for all lentivirus productions. These producer cells were transfected for lentiviral production using Lipofectamine^TM^ 2000 Transfection Reagent (Figure S6A). The viral supernatant was collected 48 and 72 h after transfection, the virus was precipitated using PEG-it™ Virus Precipitation Solution (System Bioscience, Cat No. LV810A-1) at 4 °C over the weekend and resuspended in 1/100 of the original volume. Then, 100 µL was used to transduce 750,000 HEK293T cells in a 6-well plate by addition of the viral medium 24 h after seeding. For inducible over-expression of C/EBPδ, the *CEBPD* cDNA was cloned into the *pCW57* vector (Addgene # 80921) containing a doxycycline-controlled transactivator (tTA) that binds to the TRE promoter to initiate transcription of C/EBPδ. This vector was used with the same third-generation lentiviral system as above and prepared in the same way. Then, 25 µL virus-containing medium was used to transduce 100,000 PANC-1 or MIA PaCa-2 cells in a 24-well plate. Forty-eight hours after transduction, cells were passaged and selected using 20 or 5 µg/mL Blasticidin (Invitrogen # ant-bl-05). Single cells were seeded in 96-well plates and allowed to grow out. Per cell line, a pool of three clones with high inducibility and low leakiness of C/EBPδ expression was selected for subsequent experiments.

### 4.8. Fluorescent Activated Cell Sorting

Forty-eight hours after transduction, the transduced cells were trypsinized and re-suspended in fluorescent activated cell sorting (FACS) buffer (PBS containing 1% FCS). The SONY SH800S Cell Sorter with single-cell sorting for clonogenic assays or two-way sorting to divide cells into EGFP-expression fractions was used. For MIA PaCa-2 cells, a 100 µm sorting chip was used and for PANC-1 cells, a 130 µm sorting chip was used. For RNA extraction, cells were sorted into RA1 lysis buffer (from NucleoSpin^®^ RNA-extraction Kit, Ref. 740955, Macherey-Nagel GmbH and Co. KG, Düren, Germany) and further processed for RNA extraction and quantitative RT-PCR.

### 4.9. Clonogenic Assay

Respective of the experiment, 100 cells were seeded into 24-well plates or 500 cells into a 12-well plate. Visible colonies were formed after two weeks. At this point, cells were fixed and stained using crystal violet (0.5% crystal violet in 30% ethanol/3% formaldehyde) for 10 min at room temperature followed by two washes in tap water. The colonies in 24-well plates were counted manually, only including those that count at least 50 cells. Colonies in the 12-well plate were additionally counted and measured using countPHICS [58].

### 4.10. Western Blot

Total protein was extracted by lysing cells in RIPA buffer (150 mM sodium chloride, 1% Triton X-100, 0.5% sodium deoxycholate, 0.1% SDS and 50 mM Tris (pH8.0)). Upon addition of Laemmli buffer (200 mM Tris-Cl (pH 6.8), 8% SDS, 0.4% Bromophenol blue and 40% glycerol) containing 2% 2-Mercaptoethanol, samples were fractionated by SDS/PAGE and transferred onto Immobilon-FL membranes (Millipore, Darmstadt, Germany). The blot was blocked in 5% BSA in PBS for 1 h at room temperature and incubated with primary antibodies against C/EBPδ (Santa Cruz Biotechnology, sc-365546, 1:500), eGFP (GeneTex, GTX26556, 1:5000) and α-tubulin (Santa Cruz Biotechnology, sc-23948, 1:1000) in TBS with 0.1% Tween-20 over night at 4 °C. The blot was then washed in TBS with 0.1% Tween-20, incubated with secondary HRP-linked antibodies anti-rabbit-IgG (Cell Signaling, #7074, 1:1000) or anti-mouse-IgG (DAKO, P0447, 1:2500) for 1 h at room temperature in TBS with 0.1% Tween-20, washed again and incubated with ECL Western Blotting Substrate (Pierce, #32106, Thermo Fisher) for 10 min. The blot was imaged using the ImageQuant LAS 4000 (GE Healthcare Life Sciences, Eindhoven, Netherlands).

To visualize small differences of protein expression in doxycycline-induction experiments (Figure 5D,F), these Western blots were performed using the Wes™ Simple Western capillary-based automated immunoblotting system according to the standard protocol recommended by the supplier. The resulting images were processed using the Compass software for Simple Western (ProteinSimple, San Jose, CA, USA).

### 4.11. Proliferation Assay

To assess the proliferative capacity of cell lines upon re-expression or knock-down of C/EBPδ, 10,000 cells (MIA PaCa-2 and PANC-1 transduced with a doxycycline-inducible over-expression plasmid or an empty control plasmid (*pCW57* plasmid, Addgene # 80921)) or 50,000 cells (Capan-2 transduced with shRNAs against C/EBPδ or tGFP) were seeded in 24-well plates in complete cell culture medium as described above or in the presence of 2000 ng/mL doxycycline (#D9891, Sigma-Aldrich N.V. Zwijndrecht, Netherlands) for 5 days (MIA PaCa-2 and PANC-1) and 8 days (Capan-2). Plates were scanned using the IncuCyte^®^ S3 Live-Cell Analysis System (Sartorius, Göttingen, Germany). The confluence of experimental conditions was normalized to the respective control cell line.

### 4.12. Soft Agar Tumor Sphere Formation Assay

From an autoclaved 5% agar (#30391-049, Thermo Fischer Scientific, Waltham, MA, USA) solution in saline, a 0.5% agar solution in Dulbecco’s Modified Eagle Medium (Gibco, Thermo Fischer Scientific, Waltham, MA, USA) was prepared and 800 µL was pipetted into 12-well plates to form a semi-solid bottom layer. From the same stock solution, three 0.3 % agar solutions were prepared in complete cell culture medium as described in Section 4.5 and supplemented with either no doxycycline, 100 ng/mL doxycycline or 2000 ng/mL doxycycline (#D9891, Sigma-Aldrich N.V. Zwijndrecht, Netherlands). Respectively, 6000 MIA PaCa-2 cells or 10,000 PANC-1 cells, each transduced with either a doxycycline-inducible C/EBPδ over-expression plasmid or an empty control plasmid (*pCW57* plasmid, Addgene # 80921) were added to 10 mL of 0.3% agar solution supplemented with or without doxycycline. Of the individual suspensions, 800 µL was pipetted in triplicate onto the solidified 0.5% bottom layers and left to solidify. Wells were covered with complete cell culture medium supplemented with the respective concentration of doxycycline. Fresh medium was added twice per week without doxycycline or supplemented with 200 ng/mL or 4000 ng/mL doxycycline to ensure sufficient diffusion of doxycycline to the embedded cells. MIA PaCa-2 and PANC-1 cells were allowed to form tumor spheres for two and three weeks, respectively. All wells were then scanned using the EVOS^®^ FL Cell Imaging System at 4× magnification using bright field microscopy, to capture all tumor spheres in a single plane. Spheres were then counted in Adobe Photoshop CS6 (Adobe^®^ Photoshop^®^ 2020 for Windows, San Jose, CA, USA) using the quick selection tool which was set to a diameter 23 px corresponding to 150 µm to count all spheres of this size and larger. The experiment was conducted 3 times.

### 4.13. Knock-down of C/EBPδ in CAPAN-2 Cells

To knock down C/EBPδ in Capan-2 cells, a third-generation lentiviral system using pLKO.1 puro (#8453 Addgene) containing shRNAs against CEBPD or tGFP were used. Glycerol stocks were purchased from Sigma-Aldrich (St. Louis, MO, USA) (MISSION shRNA library). We selected clones TRCN0000013695 (shCEBPD #1), TRCN0000013696 (shCEBPD #2), TRCN0000013693 (shCEBPD #3) and TRCN0000013694 (shCEBPD #4) against CEBPD and SHC004 against turboGFP as control. Bacteria were seeded on agar plates containing 100 ng/mL ampicillin and single colonies were expanded in liquid cultures. Plasmids were purified (NucleoSpin^®^ DNA, RNA and protein purification Kit, Ref. 740588.50, Macherey-Nagel GmbH and Co. KG, Düren, Germany) and incorporated in a 3rd generation lentivirus using *pMDLg/pRRE* (Addgene #12251), *pRSV-Rev* (Addgene # 12253) and *pMD2.G* (Addgene #12259). Lentiviruses were produced as described in Section 4.7 in normal HEK293T cells and Capan-2 cells were transduced in the same manner as described above and selected using Puromycin 1 µg/mL.

### 4.14. Data Analysis

Statistical analyses were performed using GraphPad Prism 6.0 (GraphPad Software Inc., La Jolla, CA, USA). Using SPSS, the data were tested for significant outliers which were excluded from further analysis. Microarray data were analyzed using paired t-tests. Immunohistochemistry data and N-status were analyzed using the Mann–Whitney U test. Kaplan–Meier survival curves were analyzed using the Mantel–Cox log rank test. C/EBPδ-expression was correlated to lymph node involvement using a one-sided Fisher’s exact test. Survival data among CEBPD-low and CEBPD-high groups were compared using the Mann–Whitney U test. For in vitro experiments, data were analyzed using the Mann–Whitney U tests whereby a one-tailed *p*-value is given for the reduction in clonal outgrowth upon C/EBPδ over-expression. Pearson correlation was used to correlate CEBPD and EGFP expression, *p*-values are one-tailed. CEBPD mRNA values of Capan-2 knock-down cell lines were tested for normality using the Shapiro–Wilk test and two-tailed *p*-values were calculated using a parametric *t*-test. Two-way ANOVA with Greenhouse–Geisser correction was used to compare growth curves. Statistical significance was set at *p* < 0.05.

## 5. Conclusions

Pancreatic ductal adenocarcinoma is the most common and most fatal form of pancreatic cancer and, to date, only few tumor suppressor genes, including *TP53, SMAD4, PTEN* and *CDKN2A,* are formally established in this context [12,13]. Here, we have identified C/EBPδ as a novel putative tumor suppressor gene that is downregulated in pancreatic ductal adenocarcinoma but not in ampullary carcinoma or intrapancreatic cholangiocarcinoma. With this discovery, we add valuable insights to the biology of pancreatic cancer and the complex context-dependent role of C/EBPδ in tumorigenesis in general, and stress that this heterogeneity should be considered in clinical practice. Maybe more importantly, we have shown that re-expressing C/EBPδ limits pancreatic cancer cell growth in a dose-dependent manner, implying that re-expressing C/EBPδ in pancreatic ductal adenocarcinoma may limit disease progression, although this remains to be established in ongoing preclinical experimental animal studies.

## Figures and Tables

**Table 1:** N-status correlates to C/EBPδ protein expression in pancreatic ductal adenocarcinoma patients.

N-Status	C/EBPδ Expression	
	Low	High
	(*N* = 47)	(*N* = 20)
N0	7	8
N1	40	12
Fisher’s exact test	*p* = 0.029	
Median survival (months)	16.9	22.2

**Table 2:** Characteristics of patients included in the cohort.

Characteristic	PDAC (*N* = 67)		AC (*N* = 42)		IP CCA (*N* = 20)	
	*N*	%	*N*	%	*N*	%
Median age (range) (years)	63 (47–83)		66.5 (48–78)		64.5 (49–82)	
Sex (M/F)	(49/18)	(73.2/26.8)	(28/14)	(66.7/33.3)	(12/8)	(60/40)
Surgery						
PPPD	59	88.1	39	92.9	18	90
Whipple–Kausch	8	11.9	3	7.1	2	10
Radicality						
R0 (≤1mm)	32	47.8	38	90.5	15	75
R1 (<1mm)	27	40.3	4	9.5	5	25
Dubious	8	11.9	0	0	0	0
Diameter post-op (cm)						
1–2	1	1.5	N/A		N/A	
2–4	34	50.7	N/A		N/A	
4–6	17	25.4	N/A		N/A	
N/A	15	22.4				
N-stage						
N0	15	22.4	27	64.3	8	40
N1	52	77.6	15	35.7	12	60
Grading						
Well differentiated	2	3	N/A		N/A	
Moderately differentiated	18	26.9	N/A		N/A	
Poorly differentiated	21	31.3	N/A		N/A	
N/A	26	38.8				
Survival						
Median (range) (months)	18.33 (1.58–88.9)		49.92 (6.6–127.38)		24.36 (1.97–103.36)	

## Supplementary Materials

The following are available online at https://www.mdpi.com/2072-6694/12/9/2546/s1. Table S1: Fold change of leading edge genes from GSEAs between CEBPD-high and CEBPD-low groups, Table S2: Pearson correlation of CEBPD mRNA with stromal scores, Figure S1: *CEBPD* mRNA correlates with stromal scores in public datasets, Figure S2: Uncropped Western blot membrane corresponding to Figure 4C, Figure S3: Uncropped Western blot membranes and WES™ Simple Western images, Figure S4: mRNA expression of CEBPD across PDAC cell lines, Figure S5: mRNA expression of putative targets of *CEBPD*, Figure S6: Establishment of lentivirus producer cells stably expressing *CEBPD*-shRNA.
