# Supplementary material for: CCAAT/Enhancer-Binding Protein Delta (C/EBPδ): A Previously Unrecognized Tumor Suppressor that Limits the Oncogenic Potential of Pancreatic Ductal Adenocarcinoma Cells
Source: Cancers (Basel). 2020 Sep 7;12(9):2546. doi: 10.3390/cancers12092546 (PMC7564797; doi:10.3390/cancers12092546)
Supplement: Supplementary file 1 [file cancers-12-02546-s001.pdf]

Supplementary Materials: CCAAT/Enhancer-Binding Protein Delta (C/EBPδ): A Previously Unrecognized Tumor Suppressor That Limits the Oncogenic Potential of Pancreatic Ductal Adenocarcinoma Cells

Leonie Hartl, JanWillem Duitman, Hella L. Aberson, Kan Chen, Frederike Dijk, Joris J.T.H. Roelofs, Mark P.G. Dings, Gerrit K.J. Hooijer, Pratika Y. Hernanda, Qiunwei Pan, Olivier R. Busch, Marc G.H. Besselink, Ton Boerman, Maikel P. Peppelenbosch, Maarten F. Bijlsma and C. Arnold Spek

Table 1. Fold change of leading edge genes from GSEAs between CEBPD-high and CEBPD-low groups. GSEAs using the PDAC dataset GSE62452 [33] and proliferative gene sets provided by Ben-Porath [35] and Chiang [36] show generally higher expression of proliferation-associated genes in patients with relatively low CEBPD mRNA expression.

BEN-PORATH [35]

Gene	Average Gene Expression in Group		CORR. *p*-Value	Fold Change (CEBPD-Low vs. CEBPD-High Group)
	CEBPD-High	CEBPD-Low		
AGFG1	7.936	8.320	1.13×10^{-4}	1.30
ANKRD27	5.743	5.981	1.67×10^{-5}	1.18
ANLN	3.580	4.299	1.97×10^{-3}	1.65
ASF1A	5.181	6.007	1.64×10^{-13}	1.77
ASPM	3.131	3.945	1.20×10^{-5}	1.76
ATAD2	4.760	5.382	1.14×10^{-6}	1.54
AVL9	5.388	5.761	3.33×10^{-9}	1.29
BUB1	3.111	3.843	4.59×10^{-7}	1.66
C1ORF112	3.125	3.717	1.09×10^{-11}	1.51
CCNB2	3.228	3.815	4.58×10^{-4}	1.50
CDCA7	3.882	4.275	1.62×10^{-3}	1.31
CDKN3	2.417	2.818	1.73×10^{-4}	1.32
CDT1	3.473	3.248	2.98×10^{-4}	0.86
CEBPG	5.834	6.031	1.95×10^{-3}	1.15
CENPE	2.162	2.384	4.11×10^{-3}	1.17
CENPF	3.665	4.513	1.99×10^{-5}	1.80
CENPM	4.493	4.123	1.76×10^{-8}	0.77
CENPN	3.823	4.114	1.05×10^{-3}	1.22
CHAF1B	4.191	4.409	3.24×10^{-4}	1.16
CKS1B	6.635	6.442	6.24×10^{-3}	0.87
CNIH4	6.130	6.433	8.68×10^{-4}	1.23
COX5A	6.875	7.148	2.84×10^{-4}	1.21
CSE1L	6.372	6.849	4.08×10^{-8}	1.39

CSNK1G1	3.814	4.061	3.08×10^{-6}	1.19
CSTB	8.575	9.086	1.40×10^{-4}	1.43
DAP3	6.886	7.217	2.56×10^{-8}	1.26
DCUN1D5	5.484	5.860	3.61×10^{-6}	1.30
DTL	3.244	3.768	6.45×10^{-5}	1.44
EXO1	3.177	3.564	1.18×10^{-3}	1.31
EZH2	4.740	5.291	7.77×10^{-9}	1.47
FAM3C	7.705	8.338	3.17×10^{-9}	1.55
FAM49B	6.410	6.914	1.53×10^{-5}	1.42
GART	6.382	6.652	2.87×10^{-6}	1.21
GNB4	5.706	6.238	1.97×10^{-3}	1.45
GTPBP4	6.469	6.837	5.75×10^{-5}	1.29
HDAC2	7.029	7.580	4.38×10^{-13}	1.47
HSPA14	4.575	4.879	6.17×10^{-8}	1.24
ILF2	7.853	8.011	7.89×10^{-3}	1.12
KDELR2	7.663	8.356	1.07×10^{-11}	1.62
LBR	7.054	7.491	6.39×10^{-7}	1.35
LGALS8	3.918	3.696	2.55×10^{-7}	0.86
MAD2L1	3.382	3.813	8.49×10^{-6}	1.35
MAGOHB	5.327	5.741	1.65×10^{-8}	1.33
MCM3	5.156	5.468	5.86×10^{-5}	1.24
MND1	2.366	2.555	6.43×10^{-4}	1.14
MSH2	4.580	5.373	2.75×10^{-16}	1.73
NDC80	3.099	3.630	2.47×10^{-6}	1.44
NDUFB5	7.051	7.456	9.21×10^{-8}	1.32
NEK2	3.427	4.021	4.33×10^{-6}	1.51
NFE2L3	4.603	4.933	6.71×10^{-4}	1.26
NMI	4.333	5.195	5.06×10^{-8}	1.82
NUDT5	4.741	5.414	1.75×10^{-12}	1.59
NUF2	3.405	3.954	2.61×10^{-4}	1.46
PARP1	6.211	6.664	1.65×10^{-8}	1.37
PBK	2.490	2.900	1.67×10^{-3}	1.33
PCNA	5.660	6.096	3.43×10^{-8}	1.35
PDCD10	4.830	5.317	3.48×10^{-8}	1.40
PDSS1	5.504	6.126	2.45×10^{-7}	1.54
PKMYT1	3.813	3.538	6.94×10^{-8}	0.83
PRC1	4.432	5.051	6.54×10^{-6}	1.54
PRIM2	3.471	3.901	1.74×10^{-8}	1.35
PRPF18	6.058	6.588	3.39×10^{-13}	1.44
PSMA3	6.250	6.837	2.63×10^{-8}	1.50
PSMB4	8.348	8.765	1.16×10^{-7}	1.33

PTS	5.488	6.062	1.67×10^{-8}	1.49
PTTG1	4.724	5.081	1.28×10^{-3}	1.28
RACGAP1	4.991	5.743	1.72×10^{-8}	1.68
RAD51	2.607	2.995	8.03×10^{-8}	1.31
RAD51AP1	3.354	3.962	9.02×10^{-7}	1.52
RANBP1	6.634	6.830	6.27×10^{-3}	1.15
RBM8A	4.949	5.527	1.89×10^{-9}	1.49
RFC4	4.683	5.400	4.70×10^{-12}	1.64
RRAGD	5.383	5.800	1.69×10^{-5}	1.34
RRM2	2.824	3.344	8.46×10^{-6}	1.43
SLC25A5	8.219	8.504	1.91×10^{-3}	1.22
SMAD2	6.829	7.245	1.24×10^{-8}	1.33
SNRPD1	4.935	5.798	1.65×10^{-10}	1.82
SNRPG	4.122	4.401	5.67×10^{-6}	1.21
SRPK1	6.478	6.825	5.53×10^{-5}	1.27
STMN1	5.451	5.855	4.57×10^{-4}	1.32
TMEM14A	5.461	6.269	4.55×10^{-14}	1.75
TOP2A	4.397	5.199	9.01×10^{-5}	1.74
TP53BP2	6.034	6.364	1.60×10^{-3}	1.26
TTK	2.246	2.704	1.20×10^{-5}	1.37
TYMS	4.626	5.472	5.07×10^{-8}	1.80
UBE2T	3.262	3.909	8.31×10^{-6}	1.57
UGGT1	7.150	7.540	5.26×10^{-11}	1.31
WDR26	7.360	7.812	1.06×10^{-8}	1.37

CHIANG [36]

**Gene**	**Average Gene Expression in Group CEBPD-High**	**CEBPD-Low**	**Corr. *p*-Value**	**Fold Change (CEBPD-Low vs. CEBPD-High Group)**
ANLN	3.580	4.299	2.59×10^{-3}	1.65
ARID3A	5.173	4.805	2.65×10^{-9}	0.77
ASPM	3.131	3.945	1.91×10^{-5}	1.76
BACE2	6.533	6.849	7.94×10^{-4}	1.25
BARD1	4.551	5.051	4.40×10^{-7}	1.41
BUB1B	3.381	3.827	2.91×10^{-4}	1.36
CCNA2	4.225	4.731	1.34×10^{-4}	1.42
CCNB1	4.415	5.122	8.01×10^{-5}	1.63
CCNB2	3.228	3.815	6.48×10^{-4}	1.50
CCNE1	4.108	4.486	5.94×10^{-7}	1.30
CDC6	3.183	3.761	3.05×10^{-4}	1.49
CDC7	3.142	3.517	3.33×10^{-4}	1.30
CDCA7	3.882	4.275	2.16×10^{-3}	1.31
CDCA7L	5.027	5.580	8.24×10^{-8}	1.47
CDKN3	2.417	2.818	2.50×10^{-4}	1.32
CENPE	2.162	2.384	5.23×10^{-3}	1.17
CENPF	3.665	4.513	3.11×10^{-5}	1.80
CENPK	3.233	3.912	2.72×10^{-5}	1.60
CEP55	3.441	3.864	1.34×10^{-3}	1.34
CKAP4	6.872	6.648	1.90×10^{-5}	0.86
CMTM3	5.787	6.176	2.25×10^{-3}	1.31
CYBA	5.056	4.590	4.52×10^{-7}	0.72
DDR1	6.177	6.463	6.04×10^{-3}	1.22

DEPDC1	2.045	2.287	7.04×10^{-3}	1.18
DEPDC1B	3.322	4.016	1.05×10^{-6}	1.62
DLGAP5	2.895	3.565	2.23×10^{-4}	1.59
DSCC1	2.769	3.254	7.27×10^{-8}	1.40
DTL	3.244	3.768	9.96×10^{-5}	1.44
DUSP9	5.718	5.318	2.94×10^{-6}	0.76
ECT2	4.525	5.357	1.38×10^{-5}	1.78
ELOVL7	6.009	6.652	9.81×10^{-6}	1.56
EZH2	4.740	5.291	1.10×10^{-8}	1.47
FANCI	4.212	4.817	7.40×10^{-6}	1.52
FBXO5	4.508	4.834	1.27×10^{-6}	1.25
FEN1	4.367	4.711	5.92×10^{-7}	1.27
FLVCR1	4.130	4.792	8.51×10^{-9}	1.58
FMNL2	6.025	6.474	2.26×10^{-5}	1.37
FUNDC1	6.137	6.648	4.45×10^{-14}	1.43
G6PD	5.501	5.201	3.63×10^{-4}	0.81
GALNT7	6.089	6.658	1.33×10^{-7}	1.48
HDAC2	7.029	7.580	9.60×10^{-13}	1.47
HELLS	3.215	3.841	2.24×10^{-7}	1.54
HMGB2	5.085	5.469	2.15×10^{-3}	1.31
IGF2BP3	3.113	3.676	2.57×10^{-3}	1.48
KIF11	2.750	3.235	1.91×10^{-4}	1.40
KIF14	2.814	3.253	1.08×10^{-3}	1.36
KIF20A	2.901	3.152	5.55×10^{-3}	1.19
KIF4A	3.284	3.626	6.45×10^{-3}	1.27
LDLRAD3	5.678	5.369	1.57×10^{-7}	0.81
LRRC1	5.798	6.266	6.66×10^{-7}	1.38
MAD2L1	3.382	3.813	1.34×10^{-5}	1.35
MAPK13	5.773	6.003	2.48×10^{-3}	1.17
MARCKSL 1	8.190	7.332	2.82×10^{-15}	0.55
MECOM	6.176	6.598	2.61×10^{-3}	1.34
MKI67	4.263	4.843	1.52×10^{-3}	1.50
MMP12	3.589	5.041	1.40×10^{-6}	2.74
MTMR2	6.458	6.855	5.00×10^{-5}	1.32
NCEH1	6.201	7.014	3.20×10^{-8}	1.76
NDC80	3.099	3.630	4.14×10^{-6}	1.44
NEK2	3.427	4.021	7.29×10^{-6}	1.51
NT5DC2	5.147	5.524	2.06×10^{-4}	1.30
NUF2	3.405	3.954	3.60×10^{-4}	1.46
NUSAP1	4.172	4.457	4.74×10^{-3}	1.22
PBK	2.490	2.900	2.26×10^{-3}	1.33
PLBD1	6.764	7.560	1.89×10^{-9}	1.74
PLP2	8.155	8.884	2.13×10^{-9}	1.66
PRC1	4.432	5.051	1.06×10^{-5}	1.54
PRKCD	6.119	6.358	5.53×10^{-3}	1.18
PRR11	3.488	3.854	2.51×10^{-3}	1.29
PTTG1	4.724	5.081	1.74×10^{-3}	1.28
RACGAP1	4.991	5.743	3.12×10^{-8}	1.68
RAD51AP1	3.354	3.962	1.53×10^{-6}	1.52
RFC4	4.683	5.400	1.08×10^{-11}	1.64
SASS6	2.906	3.408	1.39×10^{-11}	1.42
SEL1L3	7.032	7.759	1.75×10^{-11}	1.66
SHCBP1	3.709	4.441	7.05×10^{-6}	1.66
SLC38A1	7.219	7.537	1.07×10^{-3}	1.25
SLC39A10	6.062	6.459	5.58×10^{-3}	1.32
SLC7A7	4.951	5.646	8.20×10^{-6}	1.62
SMC4	5.315	5.843	1.11×10^{-5}	1.44
SPHK1	4.703	4.332	6.36×10^{-5}	0.77
SYNJ2	5.200	5.498	1.11×10^{-4}	1.23
TMEM51	4.957	5.242	2.88×10^{-3}	1.22
TMEM65	5.411	5.878	2.19×10^{-10}	1.38
TOP2A	4.397	5.199	1.36×10^{-4}	1.74
TPX2	3.414	3.901	6.14×10^{-3}	1.40
TRIP13	3.516	3.837	2.39×10^{-3}	1.25

TRNP1	6.114	5.555	5.05×10^{-11}	0.68
TSC1	5.364	5.711	3.27×10^{-8}	1.27
TTF2	4.848	5.362	2.09×10^{-9}	1.43
TTK	2.246	2.704	1.90×10^{-5}	1.37
VEGFB	6.530	6.318	5.85×10^{-4}	0.86
WASF1	3.490	3.721	1.46×10^{-3}	1.17
WSB1	8.498	8.826	6.81×10^{-4}	1.26
ZNF532	5.946	6.271	9.91×10^{-3}	1.25
ZWINT	4.379	4.573	8.26×10^{-4}	1.14

Table S2. Pearson correlation of *CEBPD* mRNA with stromal scores. Stromal gene signatures were derived from ESTIMATE analysis [44] and are correlated to *CEBPD* mRNA expression in different publicly available gene sets (Figure S1).

Gene Set	Person's r	*p*-Value
GSE15471_Badea [38]	0.4869	0.0017
GSE50827_Grimond [39]	0.2761	0.0048
TCGA_Raphael [40]	0.08705	0.2479
GSE16515_Wang [41]	0.3567	0.0327
GSE62452_Hussain [42]	0.2597	0.0312
E-MTAB-6830_Xin [43]	0.2558	0.0150

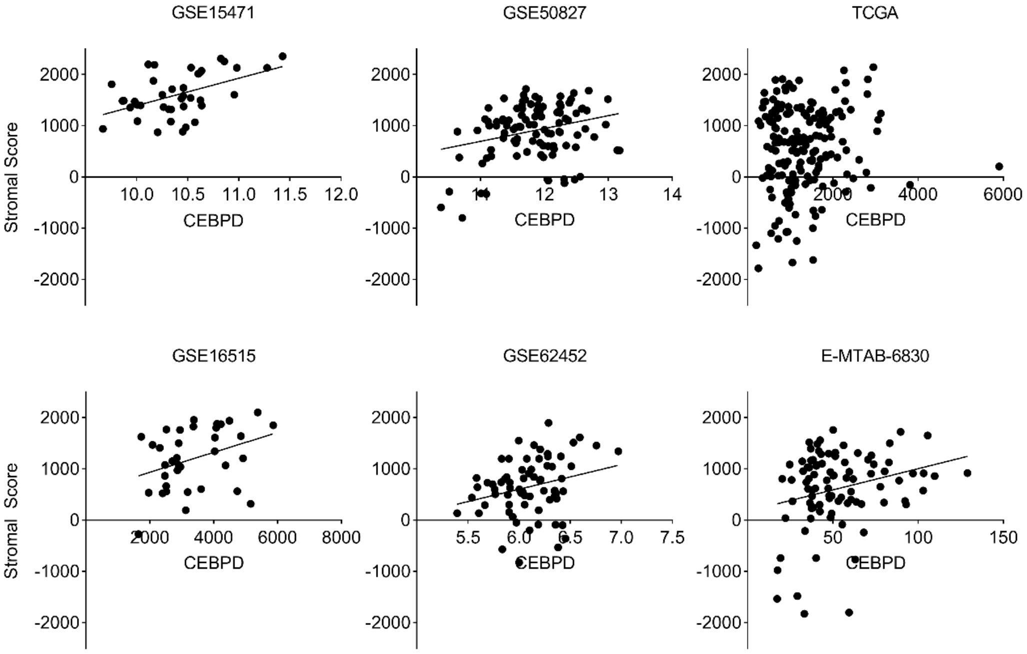

Figure S1. C/EBPδ correlates with stromal scores in public datasets. Stromal scores were calculated according to Yoshihara et al. [44] and strongly correlate with *CEBPD* mRNA expression in five out of six publicly available datasets.

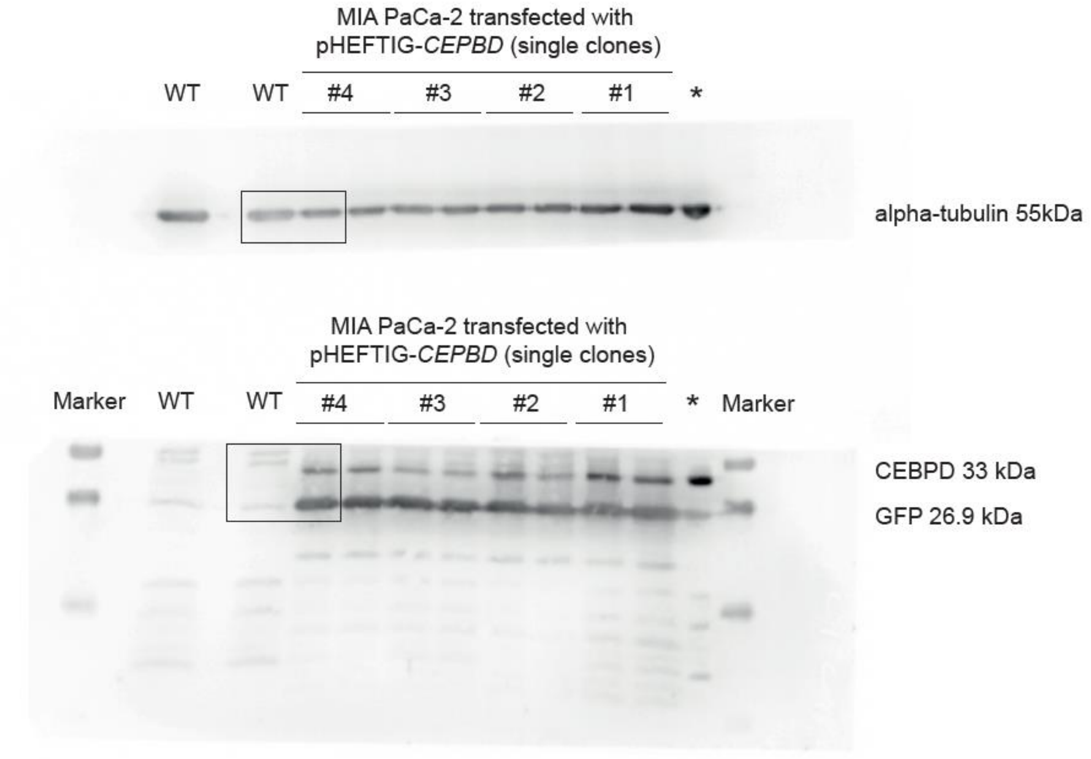

Figure S2. Uncropped Western blot membrane corresponding to Figure 4C. Black boxes indicate the samples of interest as shown in the main text.

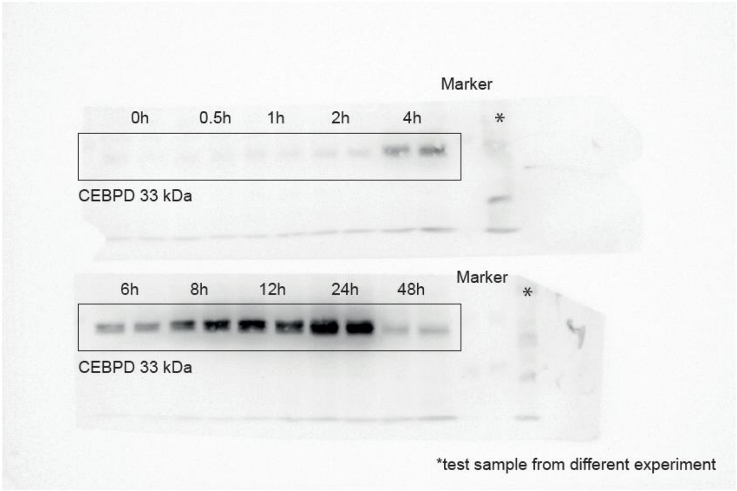

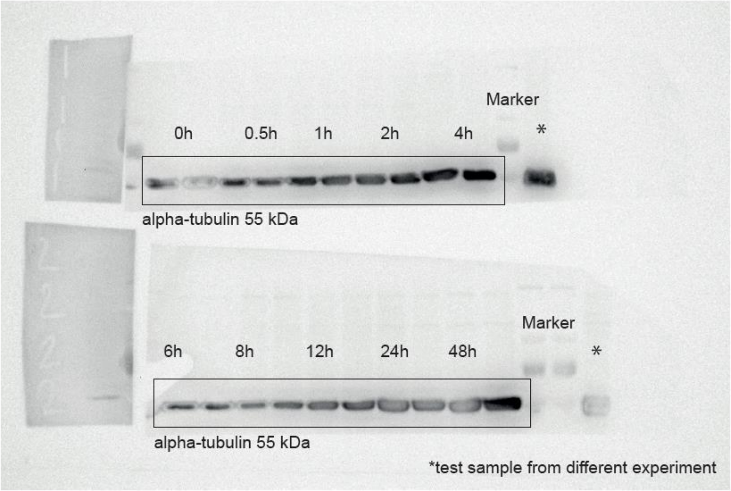
Marker
0h
0.5h
1h
2h
4h
*
alpha-tubulin 55 kDa
Marker
6h
8h
12h
24h
48h
*
alpha-tubulin 55 kDa
*test sample from different experiment

A

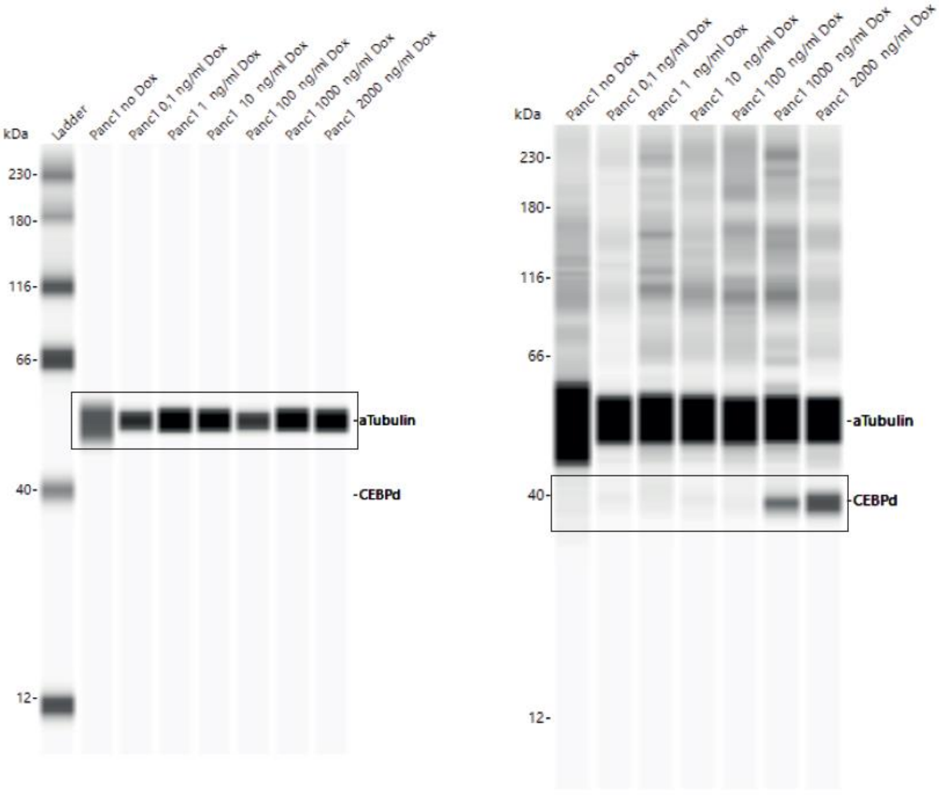
kDa
Ladder
Panc1 no Dox
Panc1 0,1 ng/ml Dox
Panc1 1 ng/ml Dox
Panc1 10 ng/ml Dox
Panc1 100 ng/ml Dox
Panc1 1000 ng/ml Dox
Panc1 2000 ng/ml Dox
230-
180-
116-
66-
40-
12-
aTubulin
CEBPd
kDa
Panc1 no Dox
Panc1 0,1 ng/ml Dox
Panc1 1 ng/ml Dox
Panc1 10 ng/ml Dox
Panc1 100 ng/ml Dox
Panc1 1000 ng/ml Dox
Panc1 2000 ng/ml Dox
230-
180-
116-
66-
40-
12-
aTubulin
CEBPd

B

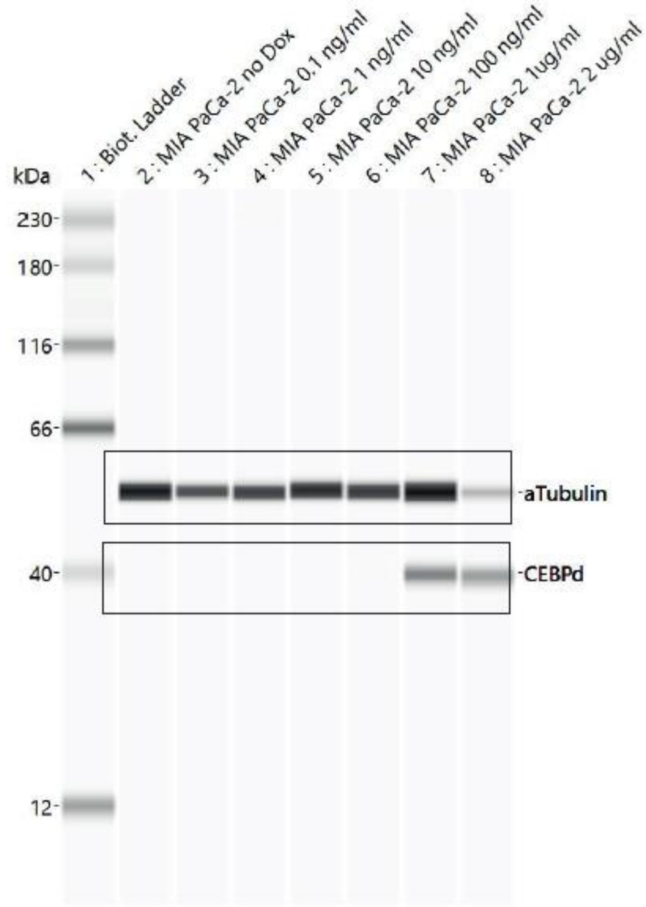

C

Figure S3. Uncropped Western blot membranes and WES™ Simple Western images. (**A**). Uncropped Western blot membrane corresponding to Figure 5B. Black boxes indicate the samples of interest as shown in the main text. (**B**). WES™ Simple Western images corresponding to Figure 5D. Both images are taken from the same samples and the same run but in the right image, the intensity was adjusted to visualize C/EBPδ in a representable manner**.** Black boxes indicate the samples of interest as shown in the main text. (**C**). WES™ Simple Western images corresponding to Figure 5F.

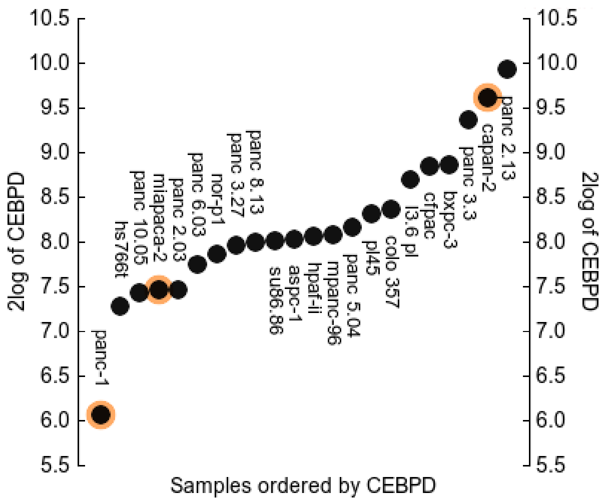

Figure S4. mRNA expression of *CEBPD* across PDAC cell lines. Data and figure are derived from Maupin et al. [46] using R2: Genomics and Visualization Platform [47].

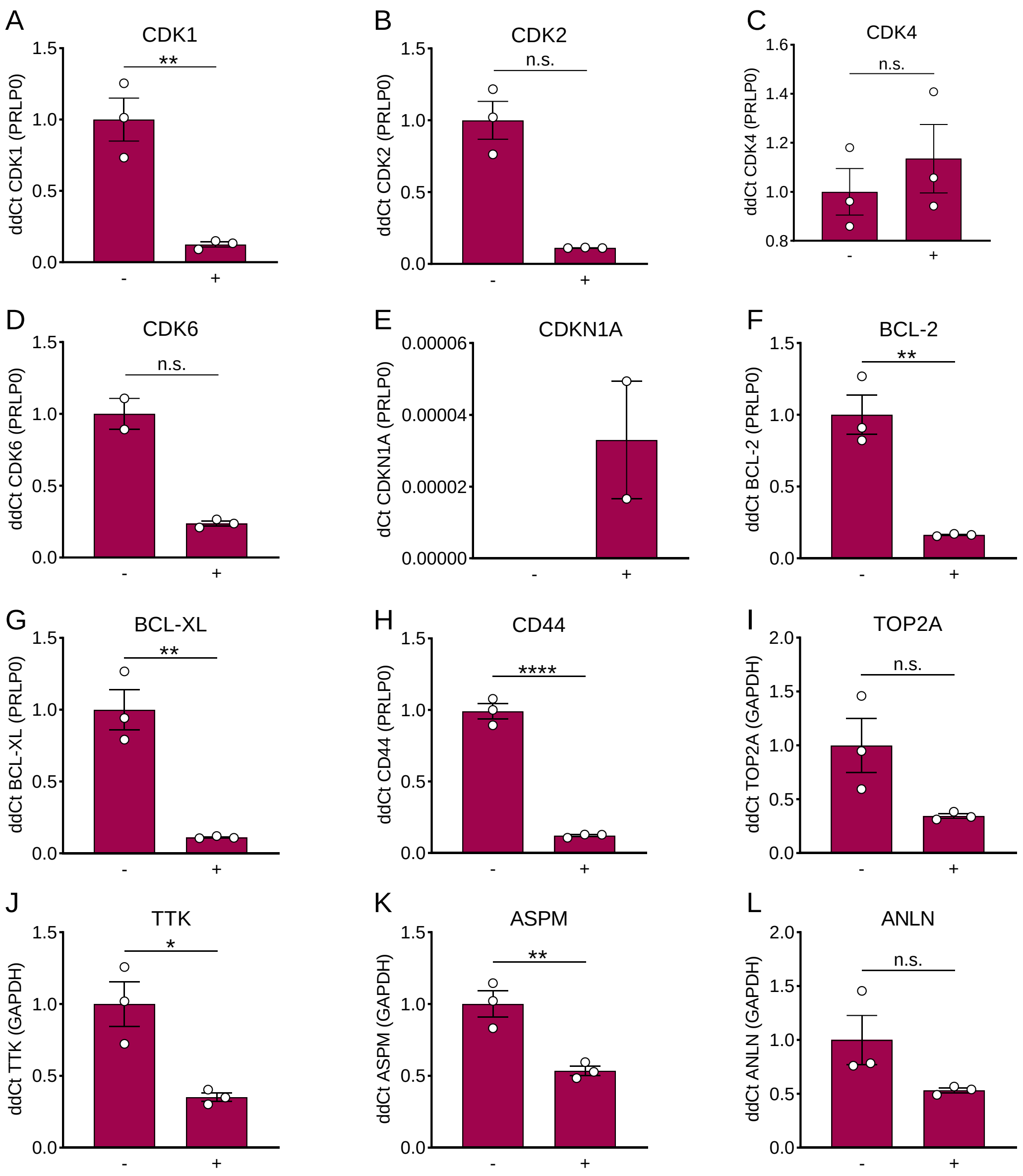

Figure S5. mRNA expression of putative targets of *CEBPD*. Bars represent MIA PaCa-2 cells inducible for *CEBPD* over-expression without (−) or with (+) doxycycine. Data are normalized to their non-induced controls. Genes are involved in: cell cycle progression (**A**–**E**), whereby CDKN1A/p21 was only detected in two samples of induced MIA PaCa-2 cells, apoptosis (**F**–**G**), stemness (H) and proliferation (**I**–**L**, from proliverative genesets by Ben-Porath and Chiang). Notbably, in MIA PaCa-2 cells C/EBPδ affects a multitude of pathways which in concurrence might account for reduced proliveration and clonogenicity of C/EBPδ-high PDAC cells. n.s.: not significant, * $p < 0.05$, ** $p < 0.01$, **** $p < 0.0001$. CDK1: Cyclin Dependent Kinase 1; CDK2: Cyclin Dependent Kinase 2; CDK4: Cyclin Dependent Kinase 4; CDK6: Cyclin Dependent Kinase 6; CDKN1A: Cyclin Dependent Kinase Inhibitor 1A; BCL-2: B-Cell Lymphoma 2 Apoptosis Regulator; BCL-XL: B-Cell Lymphoma-Extra Large; CD44: CD44 Molecule; TOP2A: DNA Topoisomerase II Alpha; TTK: TTK Protein Kinase); ASPM: Assembly Factor For Spindle Microtubules; ANLN: Anilin Actin Binding Protein.

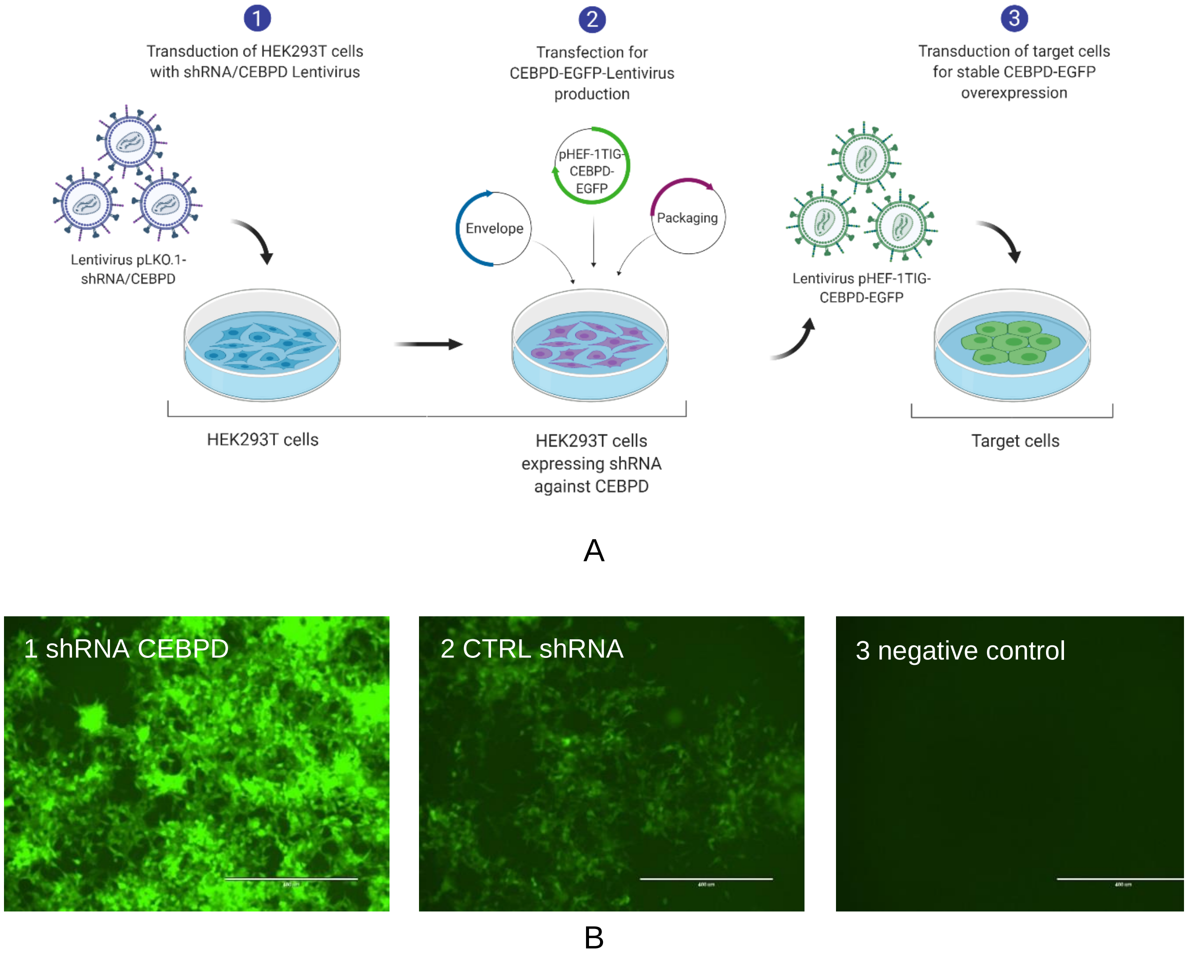

Figure S6. Knocking down *CEBPD* in lentivirus producing cells enhances virus production. (**A**) Schematic representation of pHEF-1TIG-*CEBPD*-IRES-*EGFP* lentivirus production. (1) Establishment of HEK293T cells stably expressing an shRNA against *CEBPD*. (2) Transfection of lentiviral pHEF-1TIG-*CEBPD*-IRES-*EGFP* or pHEF-1TIG-*EGFP* plasmids for virus production and collection of viral particles. (3) Transduction of HEK293T cells to determine lentivirus titer. Knocking down *CEBPD* in the producer cells has markedly enhanced lentivirus production resulting in higher virus titer, as is shown by eGFP expression in HEK293T target cells (panel B). (**B**) (1) Lentivirus titer as marked by eGFP-expression when producer cells stably expressed an shRNA against *CEBPD* (2) or a control shRNA. (3) Untransduced HEK293T cells. Scale bar is 400 µm.

References

33 Zhang, G.; Schetter, A.; He, P.; Funamizu, N.; Gaedcke, J.; Ghadimi, B.M.; Ried, T.; Hassan, R.; Yfantis, H.G.; Lee, D.H.; et al. DPEP1 inhibits tumor cell invasiveness, enhances chemosensitivity and predicts clinical outcome in pancreatic ductal adenocarcinoma. *PLoS ONE* **2012**, *7*, e31507, doi:10.1371/journal.pone.0031507.

34. Pei, H.; Li, L.; Fridley, B.L.; Jenkins, G.D.; Kalari, K.R.; Lingle, W.; Petersen, G.; Lou, Z.; Wang, L. FKBP51 affects cancer cell response to chemotherapy by negatively regulating Akt. Cancer Cell. 2009, 16, 259–266, doi:10.1016/j.ccr.2009.07.016.

35 Ben-Porath, I.; Thomson, M.W.; Carey, V.J.; Ge, R.; Bell, G.W.; Regev, A.; Weinberg, R.A. An embryonic stem cell-like gene expression signature in poorly differentiated aggressive human tumors. *Nat. Genet.* **2008**, *40*, 499–507, doi:10.1038/ng.127.

36 Chiang, D.Y.; Villanueva, A.; Hoshida, Y.; Peix, J.; Nevell, P.; Minguez, B.; LeBlanc, A.C.; Donovan, D.J.; Thung, S.N.; Sole, M.; et al. Focal Gains of Vascular Endothelial Growth Factor A and Molecular Classification of Hepatocellular Carcinoma. *Cancer Res.* **2008**, *68*, 6779–6788, doi:10.1158/0008-5472.CAN-08-0742.

38 Badea, L.; Herlea, V.; Dima, S.O.; Dumitrascu, T.; Popescu, I. Combined gene expression analysis of whole-tissue and microdissected pancreatic ductal adenocarcinoma identifies genes specifically overexpressed in tumor epithelia. *Hepato Gastroenterol*. **2008**, *55*, 2016–2027.

39 Grimont, A.; Pinho, A.V.; Cowley, M.J.; Augereau, C.; Mawson, A.; Giry-Laterriere, M.; van den Steen, G.; Waddell, N.; Pajic, M.; Sempoux, C.; et al. SOX9 regulates ERBB signalling in pancreatic cancer development. *Gut* **2015**, *64*, 1790–1799.

40 Raphael, B.J.; Aguirre, A.J. Cancer Genome Atlas Research Network. Integrated Genomic Characterization of Pancreatic Ductal Adenocarcinoma. *Cancer Cell*. **2017**, *32*, 185–203, doi:10.1016/j.ccell.2017.07.007.

42 Yang, S.; He, P.; Wang, J.; Schetter, A.; Tang, W.; Funamizu, N.; Yanaga, K.; Uwagawa, T.; Satoskar, A.R.; Gaedcke, J.; et al. A Novel MIF Signaling Pathway Drives the Malignant Character of Pancreatic Cancer by Targeting NR3C2. *Cancer Res.* **2016**, *76*, 3838–3850, doi:10.1158/0008-5472.CAN-15-2841.

43 Dijk, F.; Veenstra, V.L.; Soer, E.C.; Dings, M.P.G.; Zhao, L.; Halfwerk, J.B.; Hooijer, G.K.; Damhofer, H.; Marzano, M.; Steins, A.; et al. Unsupervised class discovery in pancreatic ductal adenocarcinoma reveals cell-intrinsic mesenchymal features and high concordance between existing classification systems. *Sci. Rep.* **2020**, *10*, e337, doi:10.1038/s41598-019-56826-9.

44 Yoshihara, K.; Shahmordgoli, M.; Martinez, E.; Vegesna, R.; Hoon, K.; Torres-Garcia, W.; Trevino, V.; Shen, H.; Laird, P.w.; Levine, D.A.; et al. Inferring tumour purity and stromal and immune cell admixture from expression data. *Nat. Commun*. **2013**, *4*, e2612, doi:10.1038/ncomms3612.

46 Maupin, K.A.; Sinha, A.; Eugster, E.; Miller, J.; Ross, J.; Paulino, V.; Keshamouni, V.G.; Tran, N.; Berens, M.; Webb, C.; et al. Glycogene expression alterations associated with pancreatic cancer epithelial-mesenchymal transition in complementary model systems. *PLoS ONE*. **2010**, *5*, e13002, doi:10.1371/journal.pone.0013002.

47 R2: Genomics Analysis and Visualization Platform. Available online: http://r2.amc.nl (accessed on 5 August 2020).

© 2020 by the authors. Licensee MDPI, Basel, Switzerland. This article is an open access article distributed under the terms and conditions of the Creative Commons Attribution (CC BY) license (http://creativecommons.org/licenses/by/4.0/).
